# Multisite musculoskeletal pain in migrants from the Indian subcontinent to the UK: a cross-sectional survey

**DOI:** 10.1186/s12891-019-2494-3

**Published:** 2019-03-28

**Authors:** E. Rizzello, G. Ntani, I. Madan, D. Coggon

**Affiliations:** 1 Department of Medical and Surgical Sciences (DIMEC), University of Bologna, Bologna, Italy; 2 MRC, Lifecourse Epidemiology Unit, University of Southampton, Southampton General Hospital, Southampton, SO16 6YD UK; 3 Arthritis Research UK/MRC Centre for Musculoskeletal Health and Work, Southampton, UK; 4 Guy’s and St Thomas’ NHS Foundation Trust, London, UK

**Keywords:** Multisite pain, Migrant, India, UK, Worker, Risk factor

## Abstract

**Background:**

Recent findings indicate that wide international variation in the prevalence of disabling regional musculoskeletal pain among working populations is driven by unidentified factors predisposing to pain at multiple anatomical sites. As a step towards identification of those factors, it would be helpful to know whether the prevalence of multisite pain changes when people migrate between countries with differing rates of symptoms; and if so, whether the change is apparent in first generation migrants, and by what age it becomes manifest.

**Methods:**

To address these questions, we analysed data from an earlier interview-based cross-sectional survey, which assessed the prevalence of musculoskeletal pain and risk factors in six groups of workers distinguished by the nature of their work (non-manual or manual) and their country of residence and ethnicity (UK white, UK of Indian subcontinental origin and Indian in India). Prevalence odds ratios (ORs) with 95% confidence intervals (CIs) were estimated by logistic regression.

**Results:**

Among 814 participants (response rate 95.4%), 20.6% reported pain at ≥3 anatomical sites. This outcome was much less frequent in Indian manual workers than among white non-manual workers in the UK (adjusted OR 0.06, 95%CI 0.01–0.36), while rates in Indian non-manual workers were intermediate (OR 0.29, 95%CI 0.12–0.72). However, within the UK, there were only small differences between white non-manual workers and the other occupational groups, including those of Indian sub-continental origin. This applied even when analysis was restricted to participants aged 17 to 34 years, and when second and later generation migrants were excluded.

**Conclusions:**

The observed differences in the prevalence of multisite pain seem too large to be explained by healthy worker selection or errors in recall, and there was no indication of bias from differences in understanding of the term, pain. Our findings suggest that whatever drives the higher prevalence of musculoskeletal pain in the UK than India is environmental rather than genetic, affects multiple anatomical sites, begins to act by fairly early in adult life, and has impact soon after people move from India to the UK.

**Electronic supplementary material:**

The online version of this article (10.1186/s12891-019-2494-3) contains supplementary material, which is available to authorized users.

## Background

Regional musculoskeletal pain, especially in the low back, neck and upper limb, is a major cause of morbidity and disability in adults of working age [1]. However, its prevalence varies substantially between countries, even among people with similar jobs [2]. Data from a longitudinal investigation in 18 countries (the CUPID study) indicate that this variation is not explained by known mechanical and psychosocial risk factors, and appears to be driven more by other, unidentified causes which predispose to pain at multiple anatomical sites [3, 4]. Across 45 occupational groups that were studied in the CUPID investigation, the prevalence of disabling low back pain at follow-up correlated with the mean number of sites other than the low back that had been painful in the 12 months before baseline (correlation coefficient = 0.58) [3]. For disabling pain in the wrist/hand, the pattern was similar, but with an even higher correlation coefficient (0.86) [4].

These findings suggest that much of the global burden of musculoskeletal pain and disability will be impervious to interventions aimed at risk factors specific to only one or two anatomical sites (e.g. heavy lifting, forceful repetitive arm movements), and that greater scope for prevention may lie in understanding what drives propensity to pain across multiple body regions, and its variation between countries. As a first step towards the identification and characterisation of underlying causes, it would be helpful to know: a) whether the prevalence of multisite pain changes when people migrate between countries with differing rates of symptoms (which would indicate that the determinants are environmental rather than genetic); and if so, b) whether the change is apparent in first generation migrants (which would imply that the drivers act soon after migration and do not depend on a long period of acculturation); and c) by what age it becomes manifest (if the change is apparent at young ages, then its causes must operate relatively early in life).

To address these three questions, we analysed data from an earlier cross-sectional survey, which assessed the prevalence of musculoskeletal pain across five anatomical sites in samples of workers from the UK and India, including some UK workers whose families came originally from the Indian subcontinent [5]. That study had already shown that rates of pain in the back, neck and arm were much lower in Indian manual workers than among manual and non-manual workers in the UK, and that office workers in India had lower rates of pain in the wrist and hand than office workers in the UK. However, it had not examined the occurrence of pain at multiple anatomical sites in the same individuals.

## Methods

The methods of data collection have been reported previously [5]. The survey focused on six groups of workers - defined by the nature of their work (non-manual or manual) and their country of residence and ethnicity (UK white, UK of Indian subcontinental origin and Indian in India). In the UK, all workers were employed by Royal Mail, either in offices (non-manual) or sorting mail by hand (manual). The non-manual workers in India were recruited from offices, including at a call centre and software house, while the Indian manual workers were employed on production lines in engineering and in the manufacture of soap and pharmaceuticals.

In each place of work, employees in relevant jobs were identified by their managers, and invited to take part in the study. Those who agreed were interviewed using a structured questionnaire, which was originally drafted in English and then translated into Marathi (with checks for accuracy by independent back-translation). The translated version was used for a subset of the Indian manual workers who preferred to be interviewed in their local language. Among other things, the questionnaire asked about demographic characteristics, mental health, occupational physical activities, psychosocial aspects of work (incentives, time pressures, support from colleagues and choice), and the occurrence in the past year of pain (lasting at least a day) at each of five anatomical sites (low back, neck, shoulder(s), elbow(s) and wrist/hand(s)).

The items about mental health were derived from the SF-36 questionnaire (with exclusion of one question that could not be translated satisfactorily) [6], and scores were graded to three levels (good, intermediate or poor) corresponding to approximate thirds of the overall distribution of scores in all subjects (i.e. with cut-points at approximate tertiles).

Physical exposures at work were ascertained by asking whether an average working day involved certain specified activities (listed in Table 1).

**Table 1:** Characteristics of participants by study group

Characteristic	UK white		UK of Indian subcontinental origin		India	
	Non-manual (*n* = 172)	Manual (*n* = 159)	Non-manual (*n* = 67)	Manual (*n* = 73)	Non-manual (*n* = 165)	Manual (*n* = 178)
Age (years)						
17–24	34 (20%)	13 (8%)	20 (30%)	19 (26%)	70 (42%)	5 (3%)
24–34	37 (22%)	42 (26%)	34 (51%)	21 (29%)	75 (45%)	23 (13%)
35–44	44 (26%)	51 (32%)	7 (10%)	24 (33%)	19 (12%)	46 (26%)
45–63	57 (33%)	53 (33%)	6 (9%)	9 (12%)	1 (1%)	104 (58%)
Sex						
Male	93 (54%)	82 (52%)	53 (79%)	45 (62%)	118 (72%)	177 (99%)
Female	79 (46%)	77 (48%)	14 (21%)	28 (38%)	47 (28%)	1 (1%)
Mental health						
Good	48 (28%)	28 (18%)	15 (22%)	18 (25%)	50 (30%)	85 (48%)
Intermediate	57 (33%)	58 (36%)	27 (40%)	18 (25%)	56 (34%)	68 (38%)
Poor	67 (39%)	73 (46%)	25 (37%)	37 (51%)	59 (36%)	25 (14%)
Occupational activities in an average working day						
Use keyboard ≥4 h	137 (80%)	4 (3%)	44 (66%)	0 (0%)	163 (99%)	3 (2%)
Other repeated movements of wrist/hand ≥4 h	4 (2%)	147 (92%)	0 (0%)	69 (95%)	23 (14%)	171 (96%)
Repeated bending/straightening of elbow for > 1 h in total	81 (47%)	140 (88%)	40 (60%)	68 (93%)	88 (53%)	163 (92%)
Work with hand above shoulder height > 1 h in total	2 (1%)	60 (38%)	1 (1%)	27 (37%)	8 (5%)	68 (38%)
Work with neck twisted > 30 min in total	19 (11%)	67 (42%)	11 (16%)	31 (42%)	40 (24%)	82 (46%)
Lifting ≥5 kg one-handed	5 (3%)	53 (33%)	4 (6%)	30 (41%)	8 (5%)	136 (76%)
Psychosocial aspects of work						
Incentives	103 (60%)	8 (5%)	50 (75%)	5 (7%)	16 (10%)	4 (2%)
Time pressure	69 (40%)	118 (74%)	23 (34%)	54 (74%)	129 (78%)	170 (96%)
Support	166 (97%)	157 (99%)	65 (97%)	72 (99%)	162 (98%)	175 (98%)
Choice	89 (52%)	112 (70%)	27 (40%)	53 (73%)	145 (88%)	32 (18%)

Regarding psychosocial aspects of work, participants were classed as being exposed to incentives if they indicated that an average working day involved either “piecework in which you are paid according to the number of articles or tasks you or your team make or finish in the day” or “payment of a bonus if you make or finish more than an agreed number of articles/tasks in the day”. Work under time pressure was defined by report that an average working day entailed “working under pressure to complete tasks by a fixed time”. Participants were deemed to receive support at work if they responded that when they had difficulties with their work, they received help and support (often or sometimes) from at least one of their colleagues, immediate superior or a trade union representative. And choice at work was defined by responses that there was often or sometimes choice in any of: “deciding how you do your work”; “deciding what you do at work”; or “deciding your work timetable or breaks”.

The questions on musculoskeletal symptoms were adapted from the modified Nordic questionnaire on musculoskeletal complaints [7], and used diagrams to illustrate the relevant parts of the body.

Participants in the UK were also asked how they would best describe their ethnic origin (White; Bangladeshi; Indian; Pakistani; Black African/Caribbean; Chinese; or other), and if non-white British, whether they were first generation, second generation, or third or more generation. Those who described themselves as Bangladeshi, Indian or Pakistani were classed as being of Indian subcontinental origin.

Full copies of the questionnaires that were used to collect data in India and the UK can be found as Additional files 1 and 2.

Statistical analysis was carried out with Stata v.12.1 software (Stata Corp LP 2012, Stata Statistical Software: Release 12.1, College Station TX, USA). We first summarised patterns of pain in the sample as a whole. Next, we compared the crude one-year prevalence of pain at different numbers of anatomical sites across the six occupational groups. We then used logistic regression to adjust the comparisons for sex, age, and other potential confounders, results being summarised by odds ratios (ORs) with 95% confidence intervals (CIs). In addition, we performed subsidiary analyses: a) restricted to participants aged < 35 years, and b) focusing on the subsets of UK workers who were first generation migrants from the Indian subcontinent. In most of the logistic regression analyses, we took UK white non-manual workers as the reference, since they provided a good representation of potentially confounding covariates. However, we also made several direct, pairwise, comparisons between other groups, in each case taking as our reference the group that had the better representation of covariates.

Ethical approval for data collection in the UK was provided by the Health and Safety Executive Research Ethics Committee. In India, the protocol for data collection was approved by the chairman of the ethics committee at the PD Hinduja National Hospital and Medical Research Centre, Mumbai, who judged that it did not need to be reviewed by the full committee.

## Results

Interviews were completed by 855 (95.4%) of the 896 workers who were invited to take part in the study, but 41 from the UK were excluded from the current analysis because they were neither white nor of Indian subcontinental origin. Table 1 summarises the distribution of the remaining 814 participants according to occupational group, demographic variables, and various potential risk factors for musculoskeletal pain. All but one of the 178 Indian manual workers were men, and they tended to be older than participants in the other occupational groups. In contrast, the non-white, non-manual workers, both in India and the UK were relatively young. As expected, prolonged use of keyboards was more frequent among the non-manual workers in both countries, while manual workers were more exposed to other sources of mechanical loading. Among the UK participants of Indian subcontinental origin, 18 (25%) of 73 manual workers and 41 (61%) of 67 non-manual workers were first generation migrants.

Table 2 shows the one-year prevalence of pain at each anatomical site in the study sample as a whole, and the frequency with which pain was reported at different numbers of sites. The highest prevalence of pain was in the low back (40.5%) followed by the neck (30.6%). Moreover, multi-site pain was common, 20.6% of participants reporting pain at ≥3 sites.

**Table 2:** One-year prevalence of pain by anatomical site and frequency with which participants reported pain at different numbers of sites

Location of pain	Number (%) of participants	
Low back	330	(40.5)
Neck	249	(30.6)
Shoulder(s)	211	(25.9)
Elbow(s)	87	(10.7)
Wrist/hand(s)	229	(28.1)
Number of anatomical sites with pain		
0	280	(34.4)
1	214	(26.3)
2	152	(18.7)
3	104	(12.8)
4	44	(5.4)
5	20	(2.5)

**Fig. 1:**
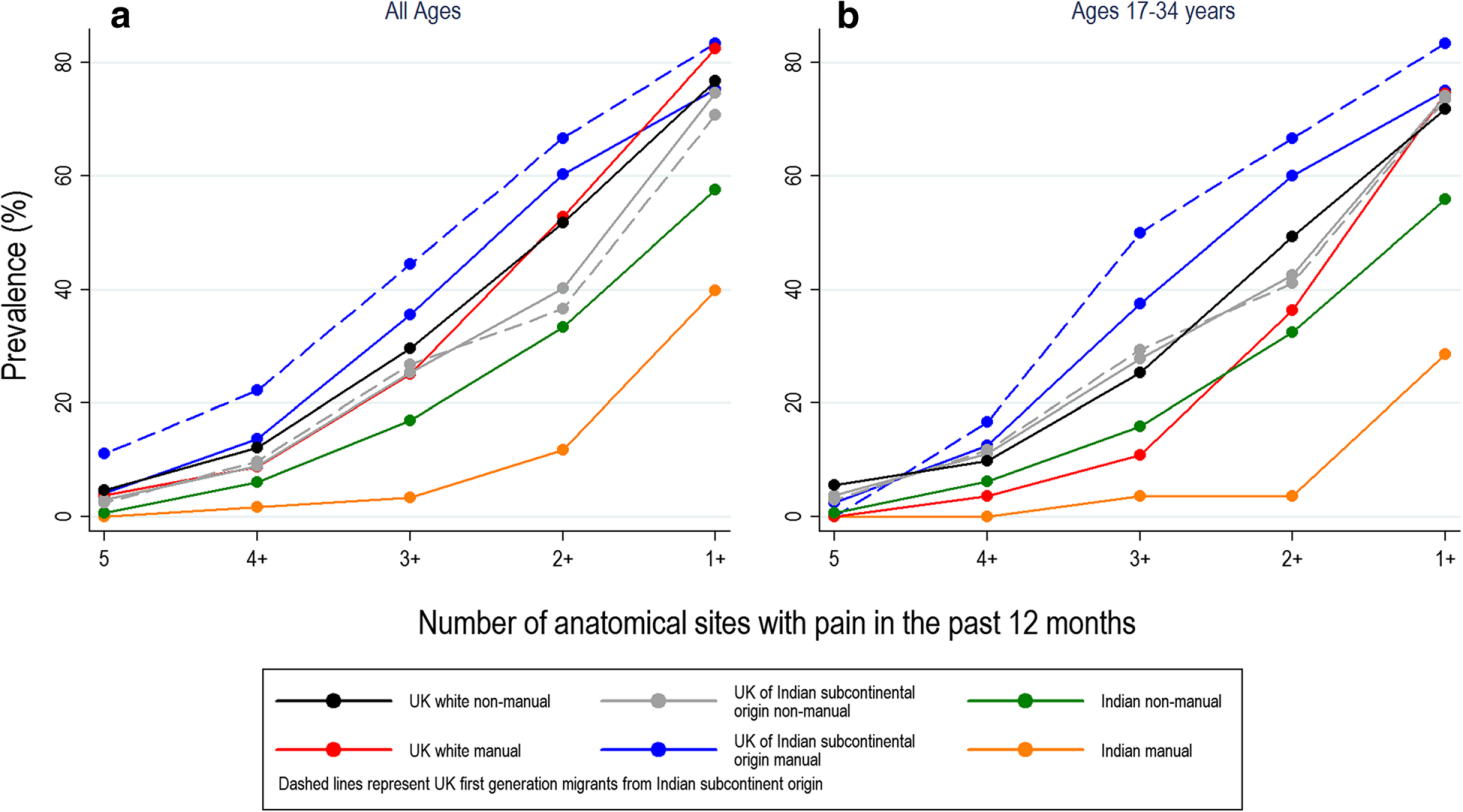
One-year prevalence of pain by occupational group and number of anatomical sites affected in all particpants (**a**) and in those aged 17-34 years (**b**)

Figure 1A) illustrates the crude prevalence of pain at multiple anatomical sites in each of the six occupational groups. Multisite pain was consistently least frequent among Indian manual workers, intermediate in Indian non-manual workers, and highest in the four occupational groups from the UK. A similar pattern was observed when analysis was restricted to participants aged 17–34 years (Fig. 1B)).

Table 3 summarises the risk of pain at different numbers of anatomical sites by occupational group, firstly after adjustment for sex and age, and then with adjustment also for the other potential risk factors listed in Table 1. For each outcome, odds ratios are relative to no pain at any site. Multisite pain was confirmed as being substantially less frequent in Indian manual workers than among white non-manual workers in the UK (fully adjusted OR for pain at ≥3 sites 0.06, 95%CI 0.01–0.36), while rates in Indian non-manual workers were intermediate, the corresponding OR being 0.29 (95%CI 0.12–0.72). Within the UK, however, there were only small and non-significant differences between white non-manual workers and the other three occupational groups, including those of Indian sub-continental origin. In direct, fully adjusted comparisons, the risk of pain at ≥3 sites in Indian manual workers was significantly lower than that among UK workers of Indian subcontinental origin, in both manual (OR 0.04, 95%CI 0.01–0.015) and non-manual (OR 0.06, 95%CI 0.01–0.39) jobs.

**Table 3:** Risk of pain in past year by occupational group according to number of anatomical sites affected

Occupational group	Number of anatomical sites with pain									
	0	≥1			≥2			≥3		
	n	N	^a^OR	(95% CI)	n	^a^OR	(95% CI)	N	^a^OR	(95% CI)
UK white – non-manual	40	132	1		89	1		51	1	
UK white – manual	28	131	^b^1.31	(0.75,2.28)	84	^b^1.24	(0.68,2.24)	40	^b^1.09	(0.56,2.11)
			^c^1.70	(0.62,4.66)		^c^1.63	(0.51,5.17)		^c^1.28	(0.30,5.42)
UK of Indian subcontinental origin – non-manual	17	50	^b^1.24	(0.63,2.45)	27	^b^1.10	(0.52,2.33)	17	^b^1.18	(0.51,2.75)
			^c^1.08	(0.52,2.23)		^c^0.90	(0.39,2.06)		^c^1.02	(0.40,2.63)
UK of Indian subcontinental origin – manual	18	55	^b^1.10	(0.57,2.11)	44	^b^1.34	(0.68,2.67)	26	^b^1.36	(0.64,2.92)
			^c^1.31	(0.44,3.93)		^c^1.70	(0.49,5.90)		^c^1.48	(0.33,6.75)
Indian – non-manual	70	95	^b^0.59	(0.36,0.98)	55	^b^0.54	(0.31,0.95)	28	^b^0.49	(0.25,0.94)
			^c^0.40	(0.21,0.77)		^c^0.37	(0.17,0.79)		^c^0.29	(0.12,0.72)
Indian – manual	107	71	^b^0.20	(0.12,0.33)	21	^b^0.09	(0.04,0.17)	6	^b^0.05	(0.02,0.12)
			^c^0.28	(0.10,0.83)		^c^0.14	(0.04,0.52)		^c^0.06	(0.01,0.36)

^a^Odds ratio (95% confidence interval) relative to no pain at any anatomical site. ^b^Adjusted for sex and age. ^c^Adjusted for all of the variables in Table 1

This pattern was maintained when analysis was restricted to younger participants (aged 17 to 34 years), with fully adjusted ORs for pain at ≥3 sites of 0.02 (95%CI 0.001–0.35), 1.15 (95%CI 0.39–3.43) and 0.44 (95%CI 0.05–3.81) respectively in Indian manual workers, UK non-manual workers of Indian subcontinental origin and UK manual workers of Indian subcontinental origin, as compared with white non-manual workers in the UK. In direct comparisons restricted to this younger age group, the risk in Indian manual workers was significantly lower than that both in UK non-manual workers of Indian subcontinental origin (fully adjusted OR 0.01, 95%CI 0.001–0.29) and in UK manual workers of Indian subcontinental origin (OR 0.04, 95%CI 0.003–0.42).

Furthermore, the similarity of risk in UK workers of Indian subcontinental origin to that in white UK workers was still apparent when second and later generation migrants were excluded (ORs for pain at ≥3 sites relative to UK white non-manual workers 1.80, 95%CI 0.27–11.96 for first generation migrants in manual work and 0.80, 95%CI 0.28–2.31 for first generation migrants in non-manual work). In direct comparisons, the risk of pain at ≥3 sites in Indian manual workers was significantly lower than that of first generation migrant workers in the UK, both in manual (fully adjusted OR 0.04, 95%CI 0.01–0.23) and non-manual jobs (fully adjusted OR 0.09, 95%CI 0.01–0.65).

## Discussion

Our analysis indicates that in the working populations studied, multisite musculoskeletal pain was substantially more common in the UK than in India. However, among workers in the UK of Indian sub-continental origin, including first generation migrants and those aged < 35 years, rates of pain were close to those of their white colleagues. These results suggest that whatever drives the much higher prevalence of musculoskeletal pain in the UK than India is environmental rather than genetic, affects multiple anatomical sites, begins to act by early in adult life, and has impact fairly soon after people move from India to the UK.

Our study benefited from a high response rate among those eligible to take part. Moreover occupational exposure to physical activities was (by design) much the same among the three groups of manual workers, and rather lower among the three groups of non-manual workers [5]. Differences in the physical demands of work are therefore unlikely to explain the lower prevalence of pain in Indian manual workers.

It is possible that some workers with disabling musculoskeletal pain were excluded from the sampling frame because they were absent from work at the time of the survey or had been forced to leave their jobs, and that this healthy worker selection was stronger in India than in the UK. However, we think it is unlikely that any resultant bias could explain such large differences in pain prevalence as were observed. For that to occur, well over half of all men taken on to work in the Indian manual jobs would have to leave their jobs because of musculoskeletal pain.

An earlier report, based on the same study, described the prevalence of pain in the past month at specific anatomical sites [5]. However, in the current analysis, we focused on the one-year prevalence of symptoms since it was expected to provide a more sensitive measure of general propensity to pain, and for that reason had been used previously in the CUPID study [3, 4]. For this purpose it did not matter whether the pain at different anatomical sites had occurred simultaneously – only the number of sites that had been affected at some time during the period of interest. Recall of pain over the longer period may not have been as accurate as that for the past month. However, we know from the earlier analysis that in comparison with participants in the UK, the Indian workers, and especially those in manual jobs, also had a lower one-month prevalence of pain in both the low back and arm [5]. It therefore seems unlikely that the differences we observed in the one-year prevalence of multisite pain can be attributed to errors in recall.

Ideally, as in the CUPID study, our assessment of the extent of pain would have distinguished between upper limb pain affecting the right and left sides of the body. However, the questionnaire that had been used when interviewing workers in India had not asked about the laterality of pain in the elbow and wrist/hand, and therefore it could not be done.

Another possible source of bias was differences in understanding of the term, pain, especially when the questionnaire was translated into Marathi. However, among the Indian manual workers, the prevalence of pain was much the same, whether interviews were in Marathi or English (data available on request).

In the logistic regression analyses for Table 3, we took UK white, non-manual workers as our reference since they provided reasonable representation across the distribution of covariates. However, we did also make direct pairwise comparisons between other groups of workers. Importantly, the risk of pain at ≥3 sites among Indian manual workers was significantly lower, not only than that in the main reference group, but also than that in UK manual and non-manual workers of Indian subcontinental origin. The subsidiary analyses for younger workers and those who were first generation migrants to the UK included fewer participants, and were therefore subject to greater statistical uncertainty. Nevertheless, again with pain at ≥3 sites as the outcome, the risk for Indinan manual workers was significantly lower than that in UK manual and non-manual workers who were first generation migrants from the Indian subcontinent. And among participants aged < 35 years, risk among Indian manual workers was significantly lower than for UK manual and non-manual workers of Indian subcontinental origin.

The levels of pain prevalence that we found in our study cannot be compared directly with those in other surveys that have been based in the general population rather than workers in employment, covered different age ranges, and used different outcome measures (e.g. chronic widespread pain). However, our finding that rates of pain were lower in India than in the UK is consistent with the CUPID study, in which prevalence was lower in Pakistan and Sri Lanka than in the UK [2–4]. Moreover, as in the CUPID investigation, the differences applied to musculoskeletal pain in general and were not limited to just one or two anatomical sites. It is striking, however, that rates of pain among UK workers of Indian subcontinental origin were close to those of their white colleagues. Furthermore, this appeared to apply also to the subset of first generation migrants, and when analysis was restricted to younger ages (< 35 years).

We have been unable to identify any other studies that used standardised methods to compare the prevalence of musculoskeletal pain in migrant populations with those both in their country or region of origin and in the country to which they had moved. However, a survey in the North-West of England found that pain in the past month lasting > 1 week was slightly more common among migrants from South Asia than in the local white population, while “pain in most joints” lasting > 1 week in the past month was substantially more frequent [8]. A second study, which recruited also in the West Midlands, similarly found a higher prevalence of widespread pain (“all over the body in the past month”) in South Asian ethnic groups than in white Europeans [9]. And in a more recent survey carried out in the Tower Hamlets district of London, the prevalence of chronic widespread pain (in two contralateral quadrants of the body and also the axial skeleton and present for at least three months) was greater among people of Bangladeshi origin than in white British/Irish participants [10]. While these investigations differed from ours in the pain outcomes that were examined and the demographics of the populations studied, they support the view that rates of pain in South Asian migrants to the UK are at least as high as in the indigenous white population.

The pattern of results in our study implies that the drivers of the large differences in musculoskeletal pain between workers in India and the UK are environmental, and predispose to pain at multiple anatomical sites. It suggests, moreover, that they act early in the lifecourse, and begin to affect migrants fairly soon after they move from India to the UK. Beyond this, our data do not indicate what the drivers might be, but one possibility is that awareness of musculoskeletal pain and responses to it are importantly influenced by a person’s social environment, in the same way that a number of other illnesses appear to be culturally determined [11]. If so, rates of musculoskeletal pain would be expected to decline when people migrate from countries with high prevalence (e.g. in South and Central America) to places where it is less frequent (e.g. in Europe) – a hypothesis that would be worth testing in future research.

## Conclusions

Whatever drives the much higher prevalence of musculoskeletal pain in the UK than the Indian subcontinent is environmental rather than genetic, affects multiple anatomical sites, and appears to act by early in adult life, with impact fairly soon after people move from the subcontinent to the UK.

## Additional files

Additional file 1: India Questionnaire. **Description of data:** Copy of questionnaire used to collect data in India (DOCX 307 kb)

Additional file 2: UK Questionnaire. **Description of data:** Copy of questionnaire used to collect data in UK (DOCX 336 kb)

## Abbreviations

CI: confidence interval
CUPID: Cultural and psychosocial influences on disability
OR: odds ratio
SF-36: Short Form-36
