# Supplementary figures and images for: Multisite musculoskeletal pain in migrants from the Indian subcontinent to the UK: a cross-sectional survey
Source: BMC Musculoskelet Disord. 2019 Mar 28;20:133. doi: 10.1186/s12891-019-2494-3 (PMC6440109; doi:10.1186/s12891-019-2494-3)

**India Questionnaire**


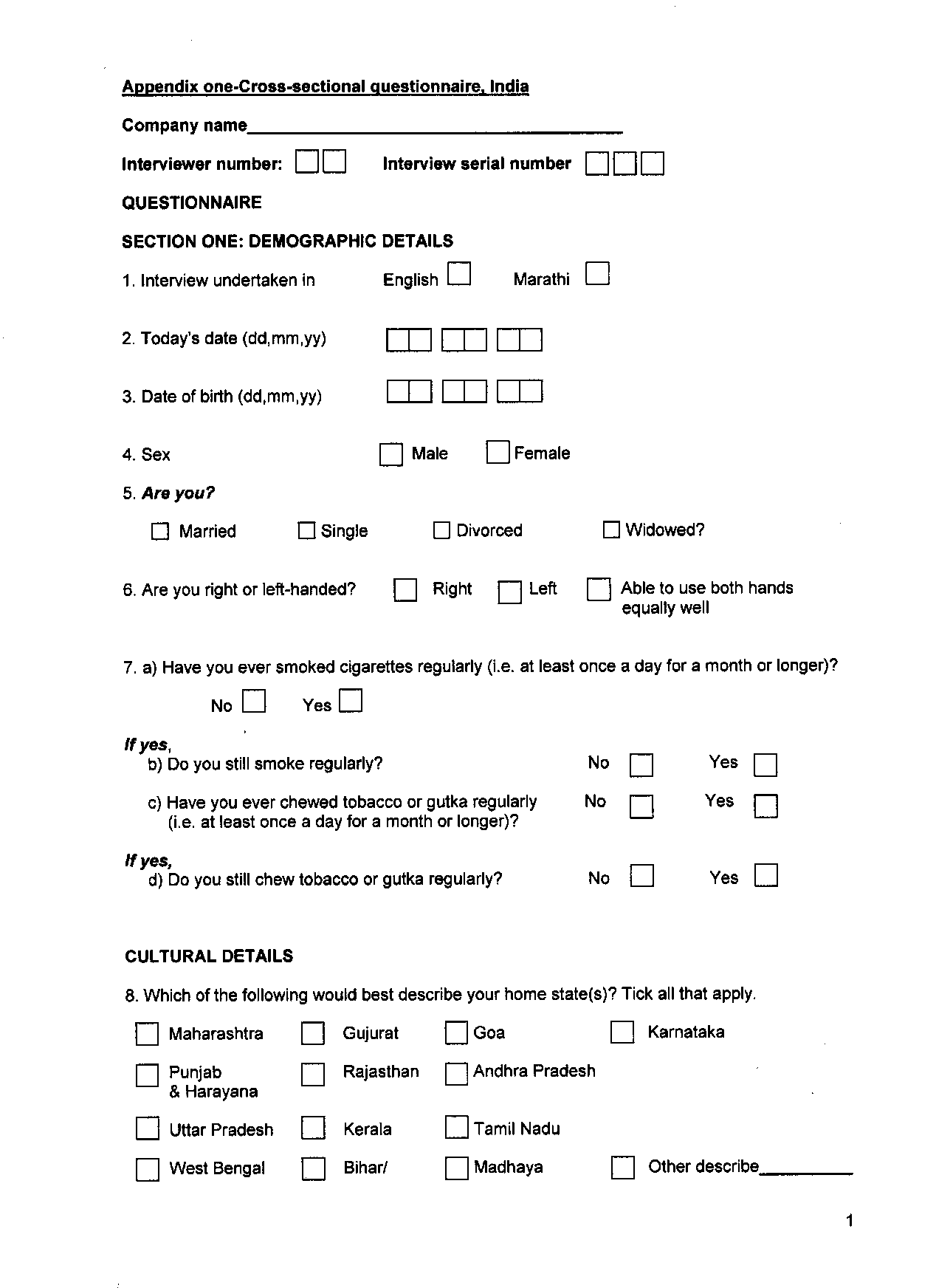

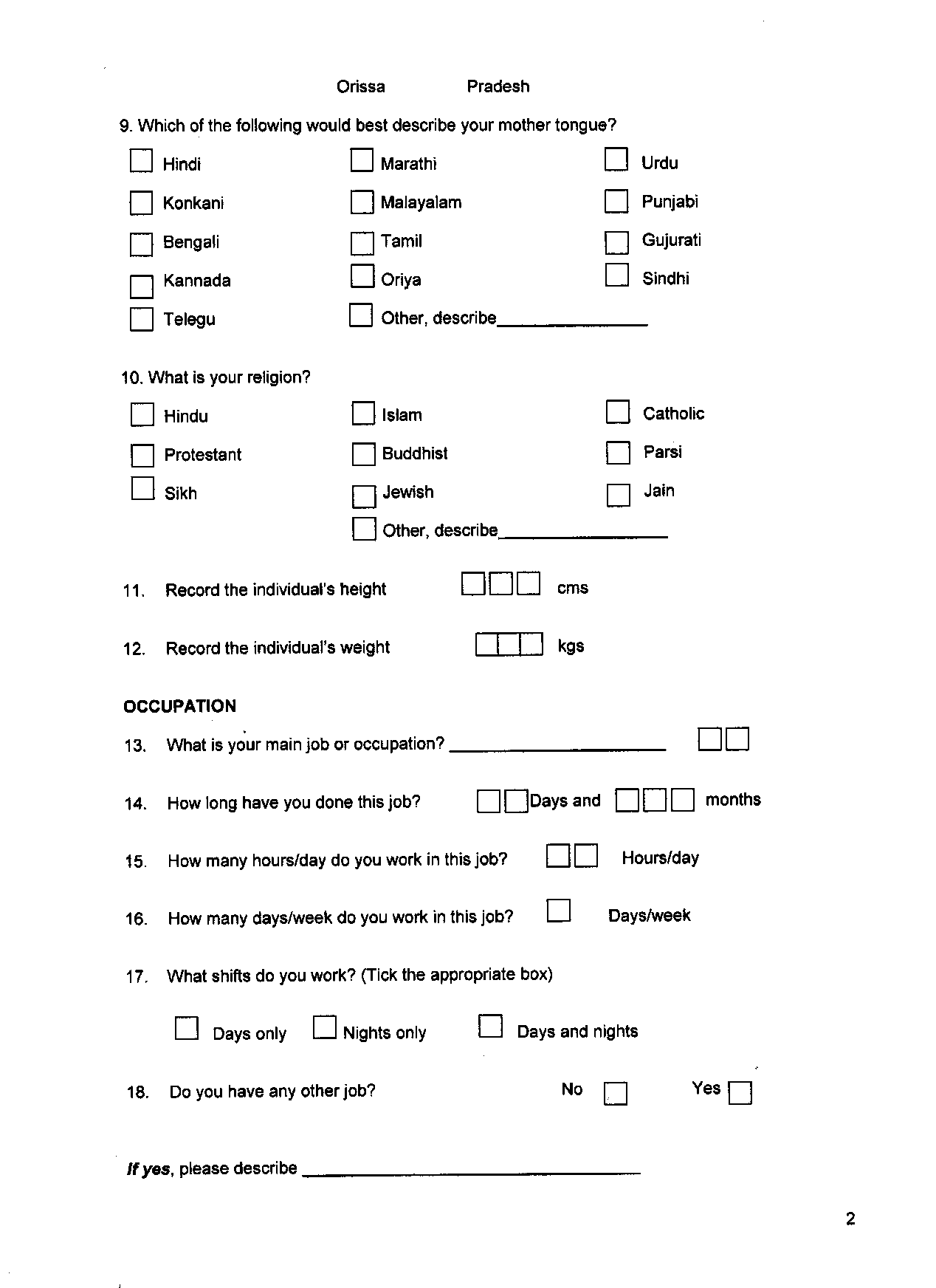


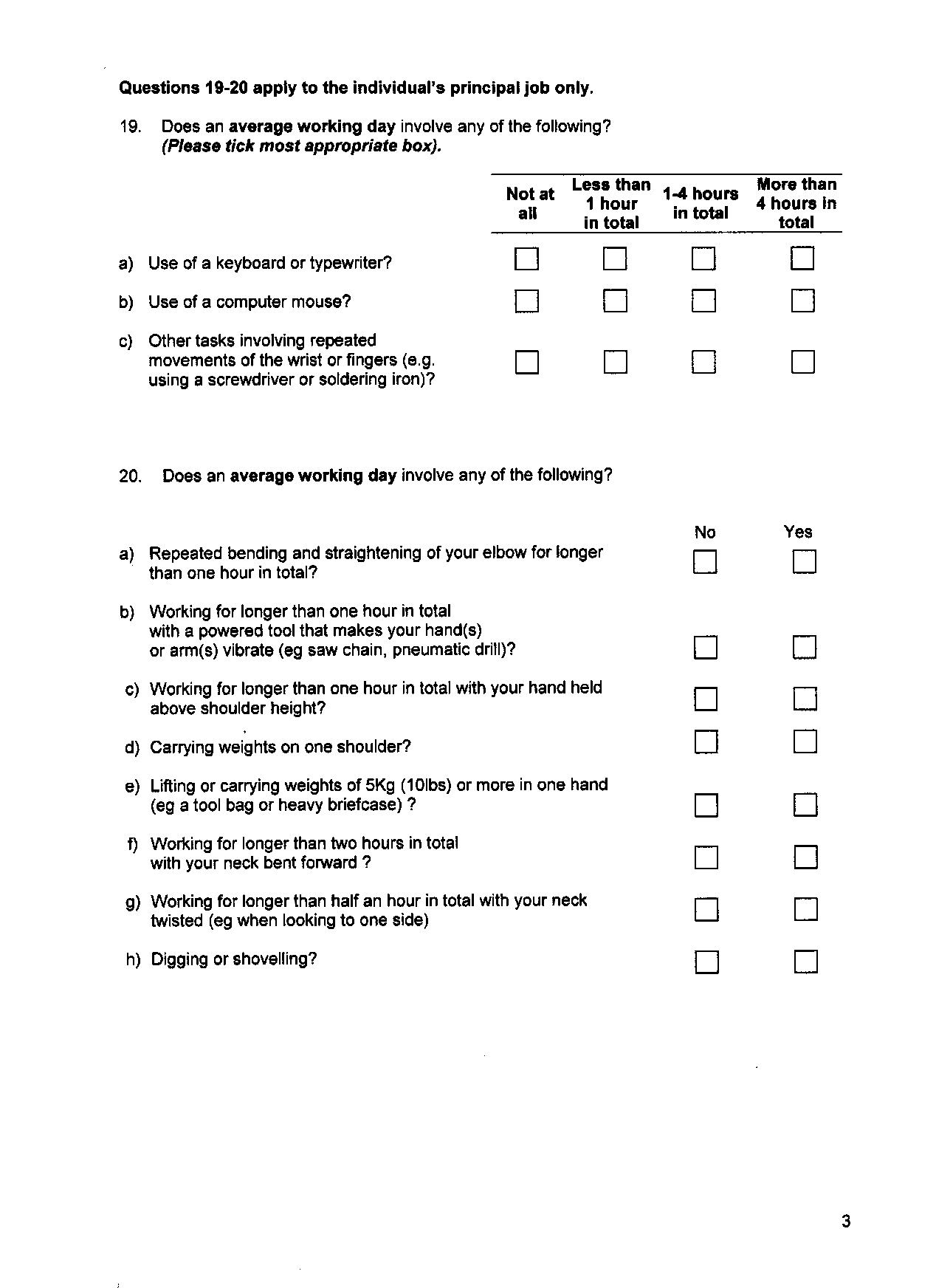


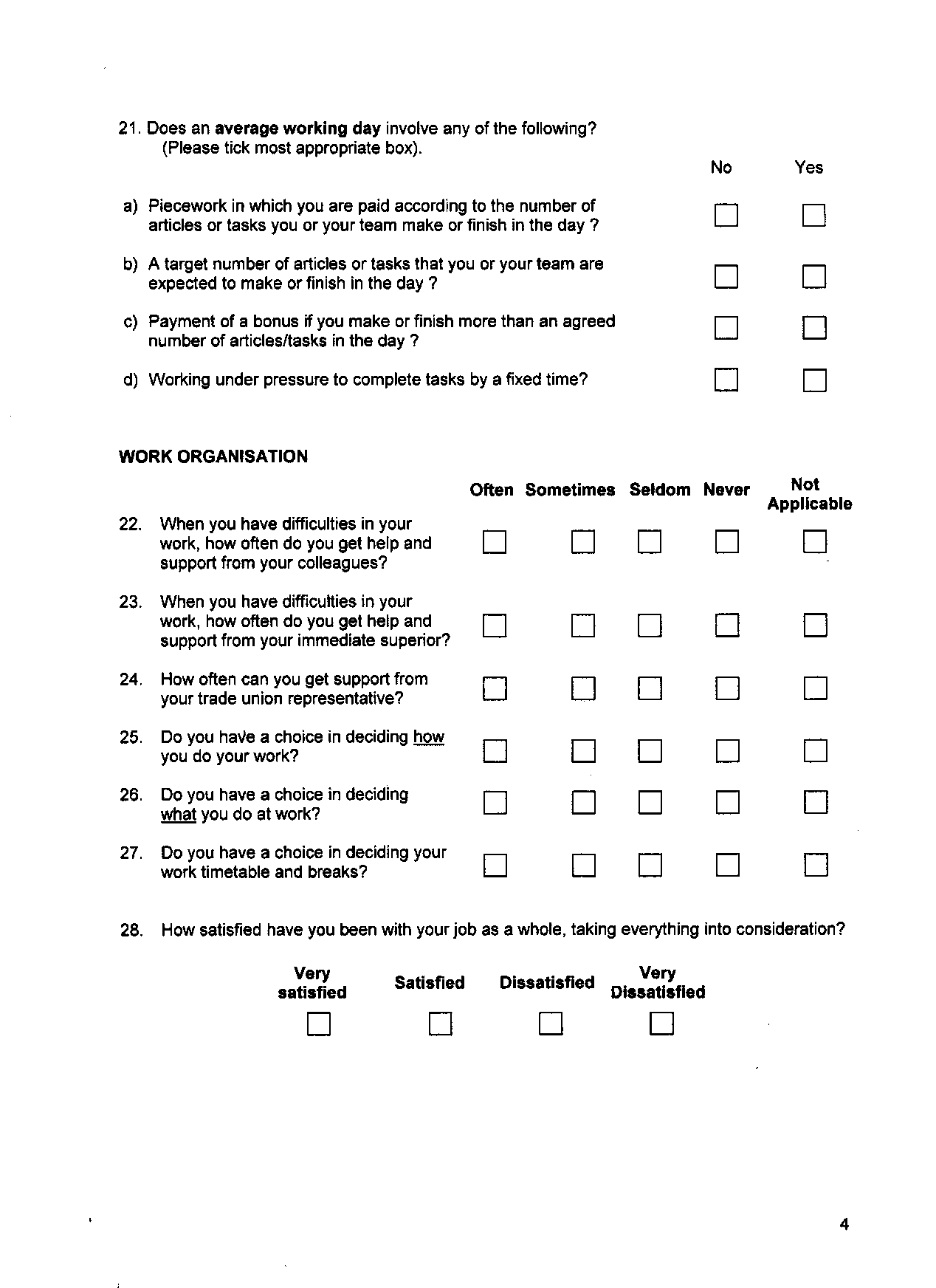


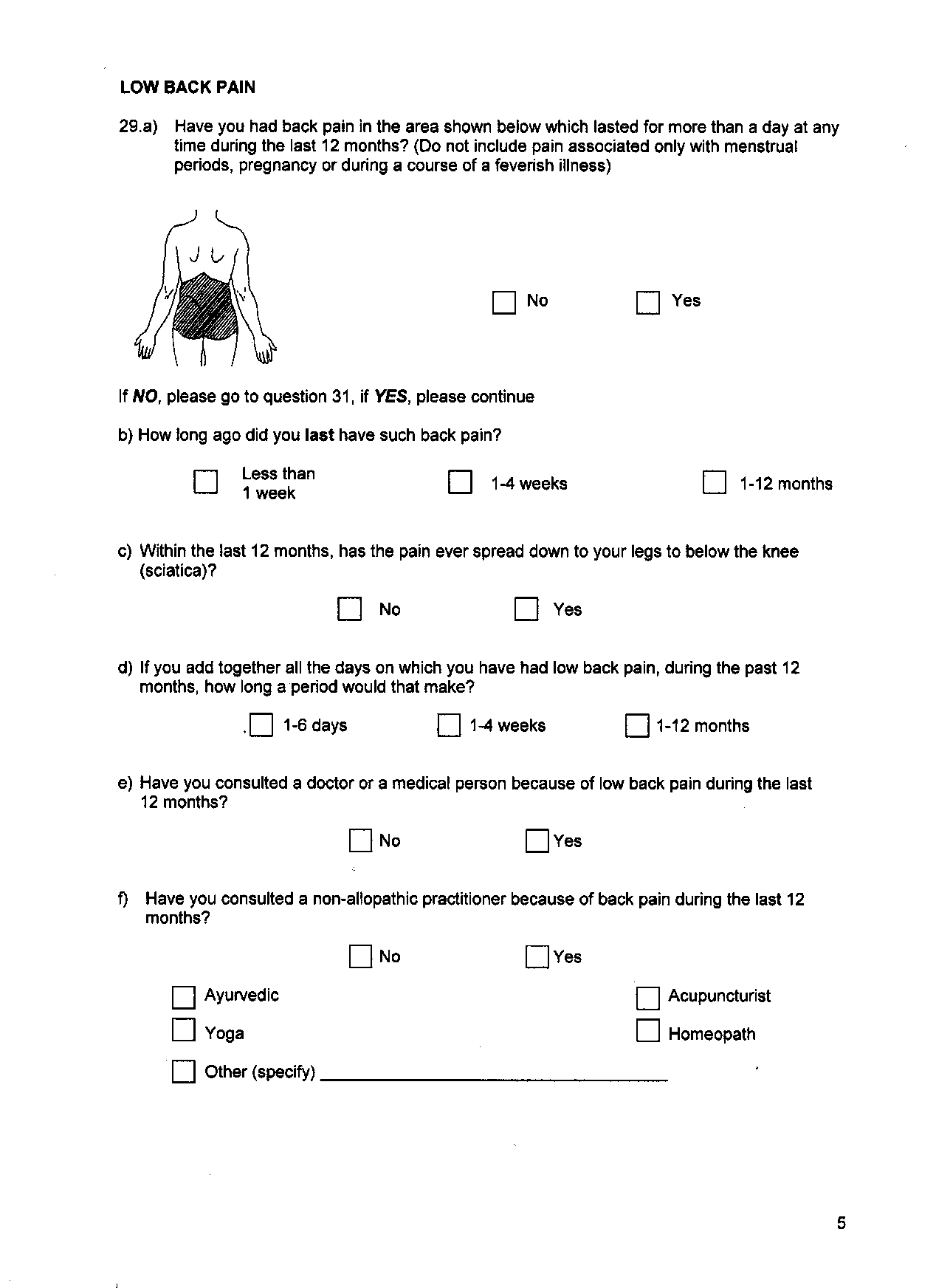


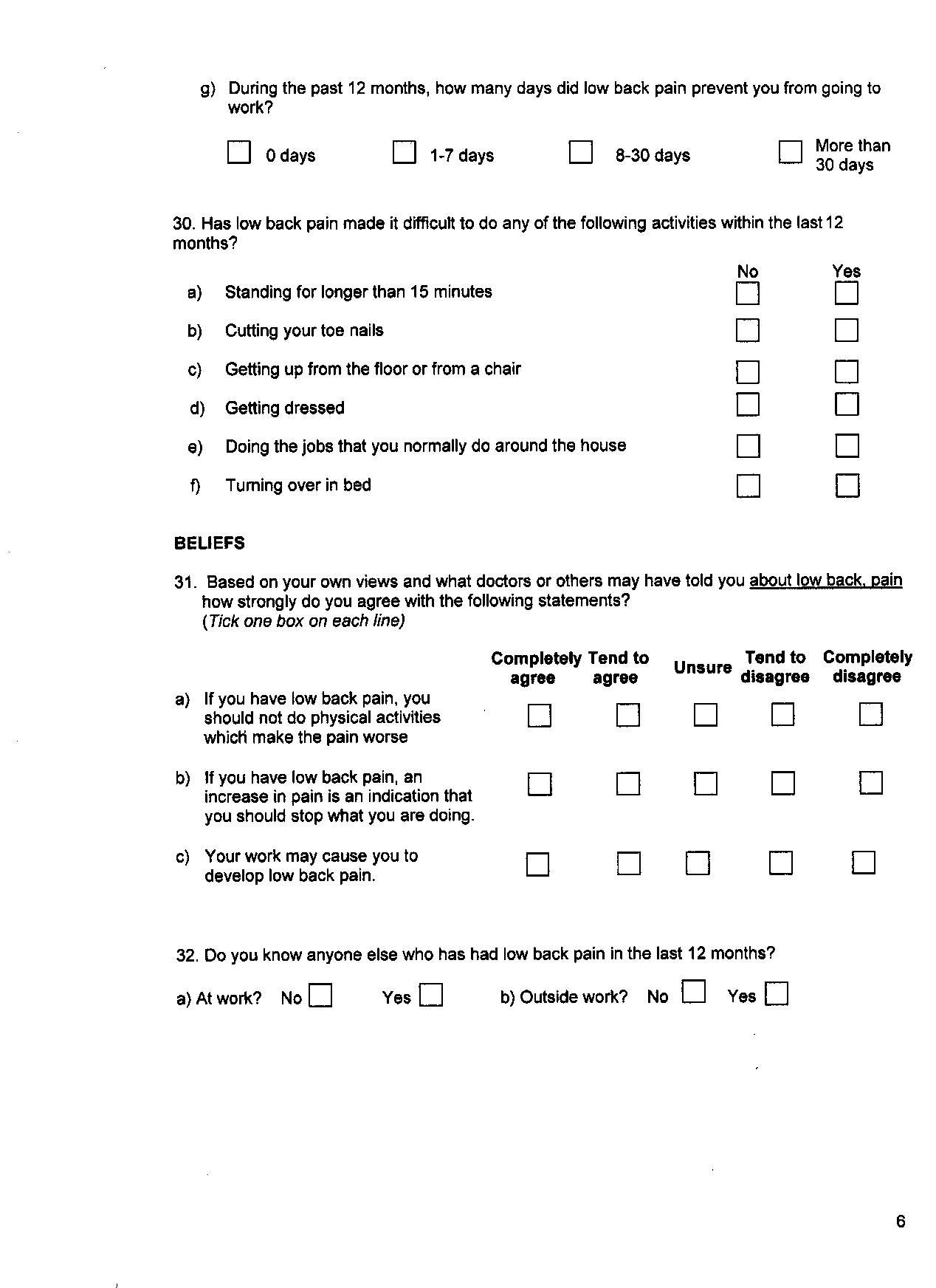


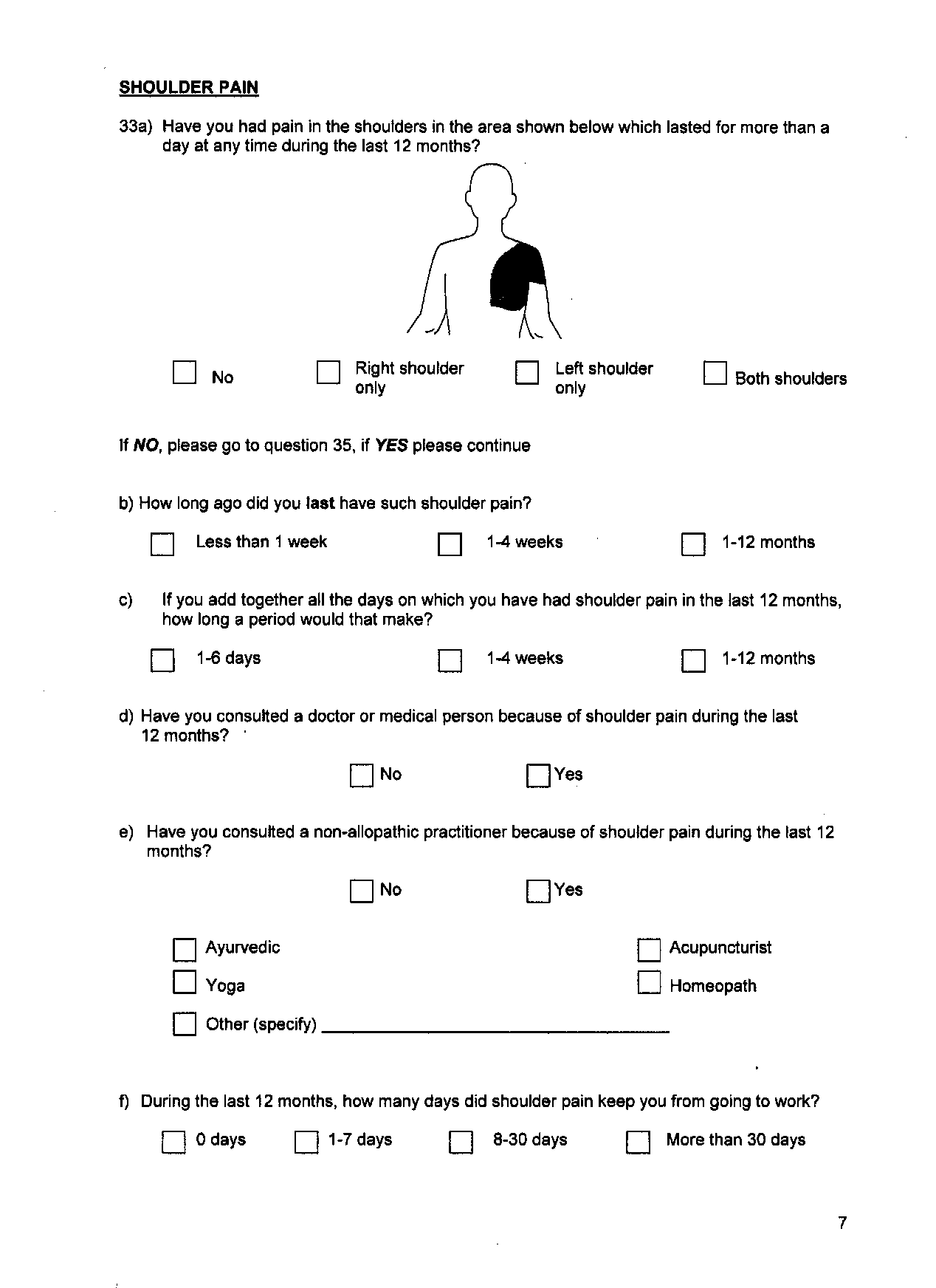


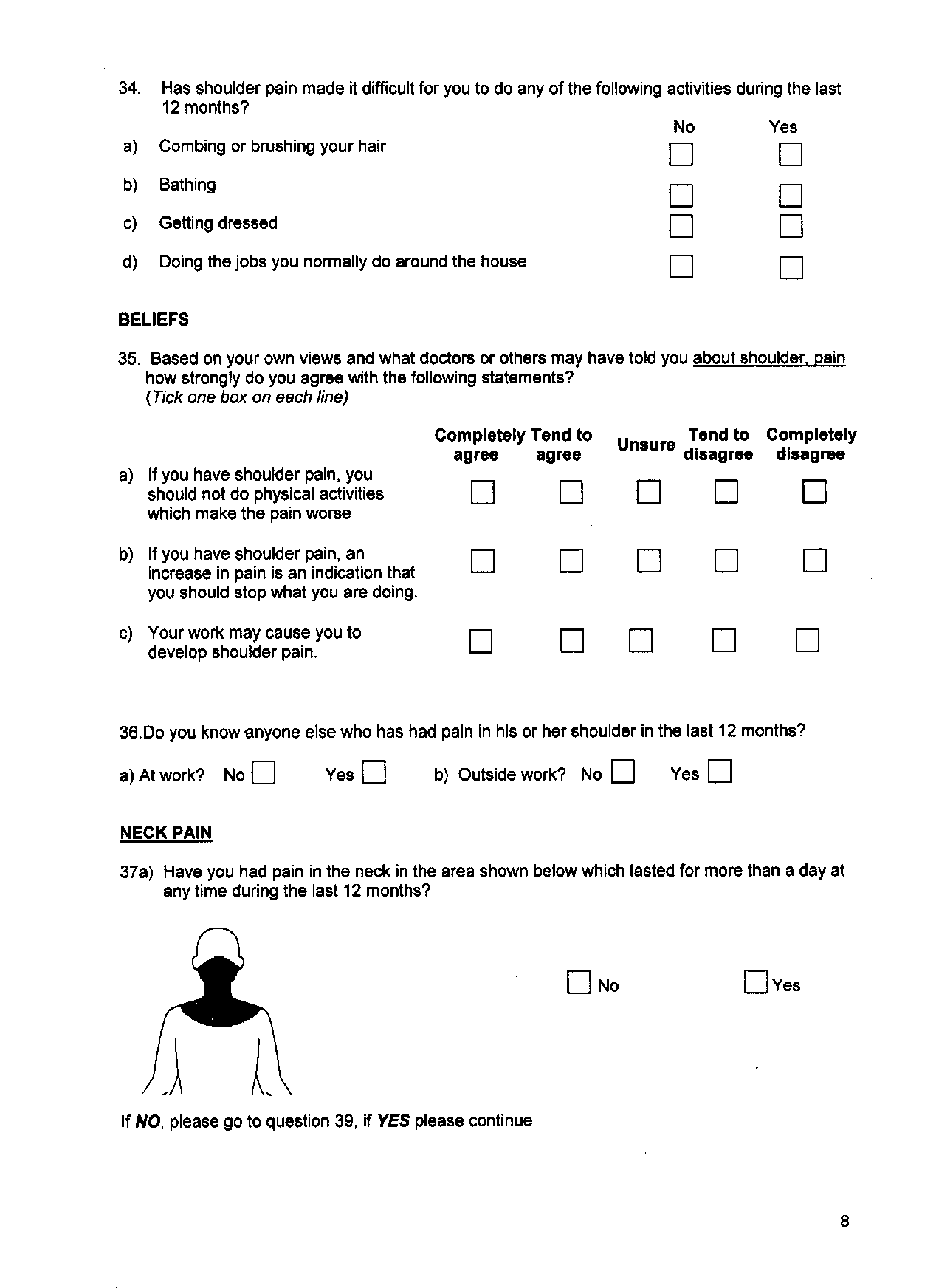


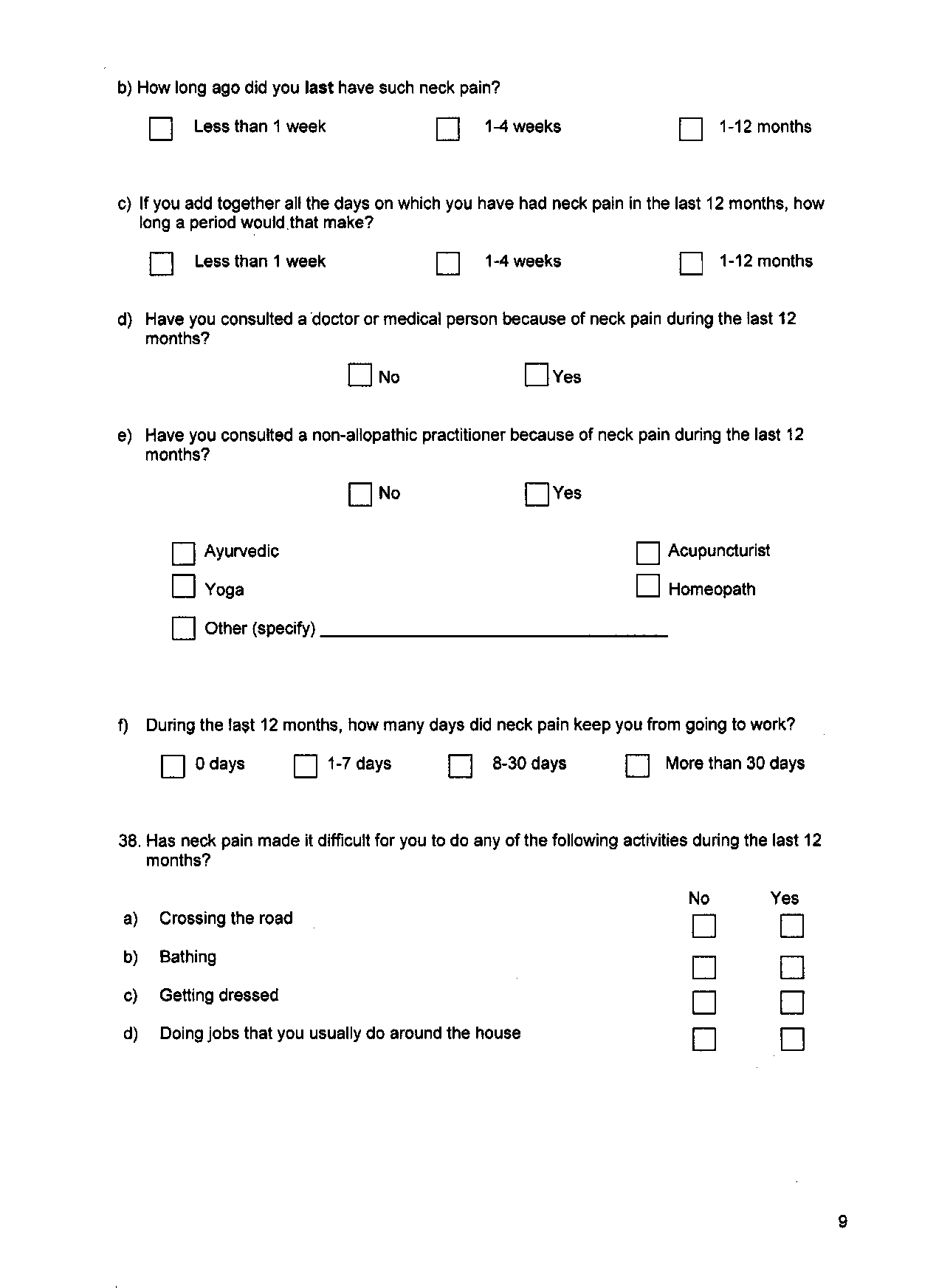


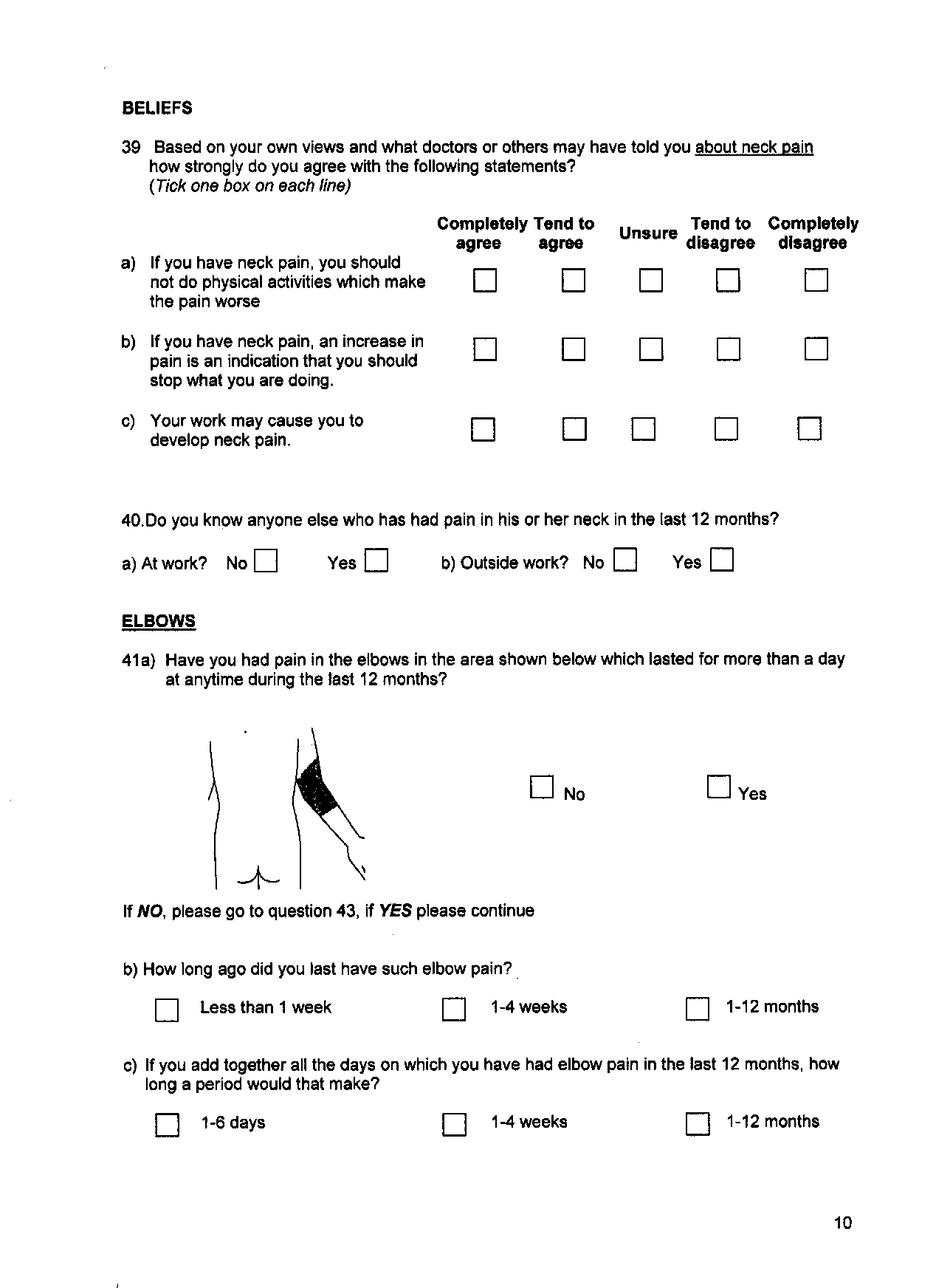


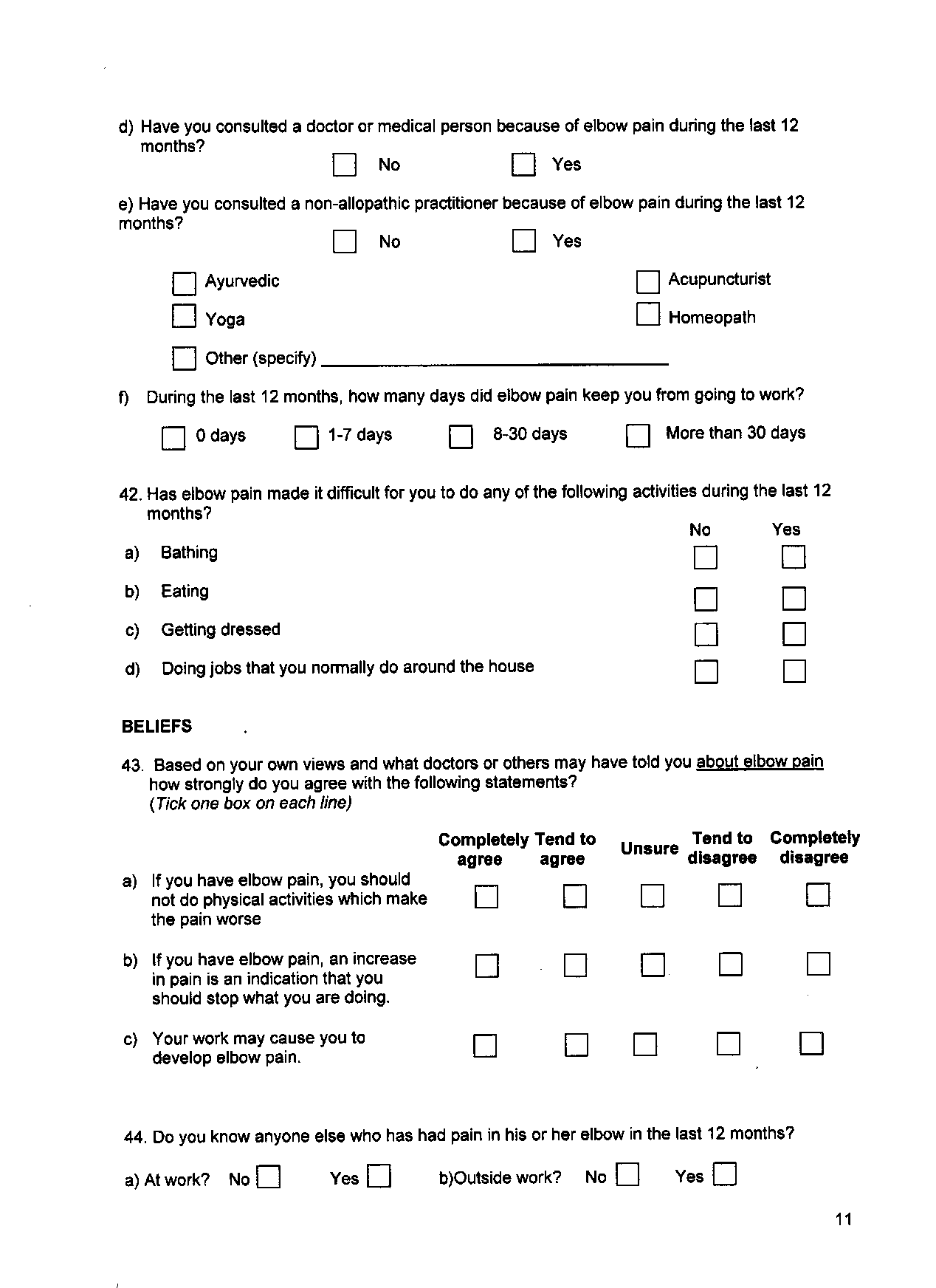


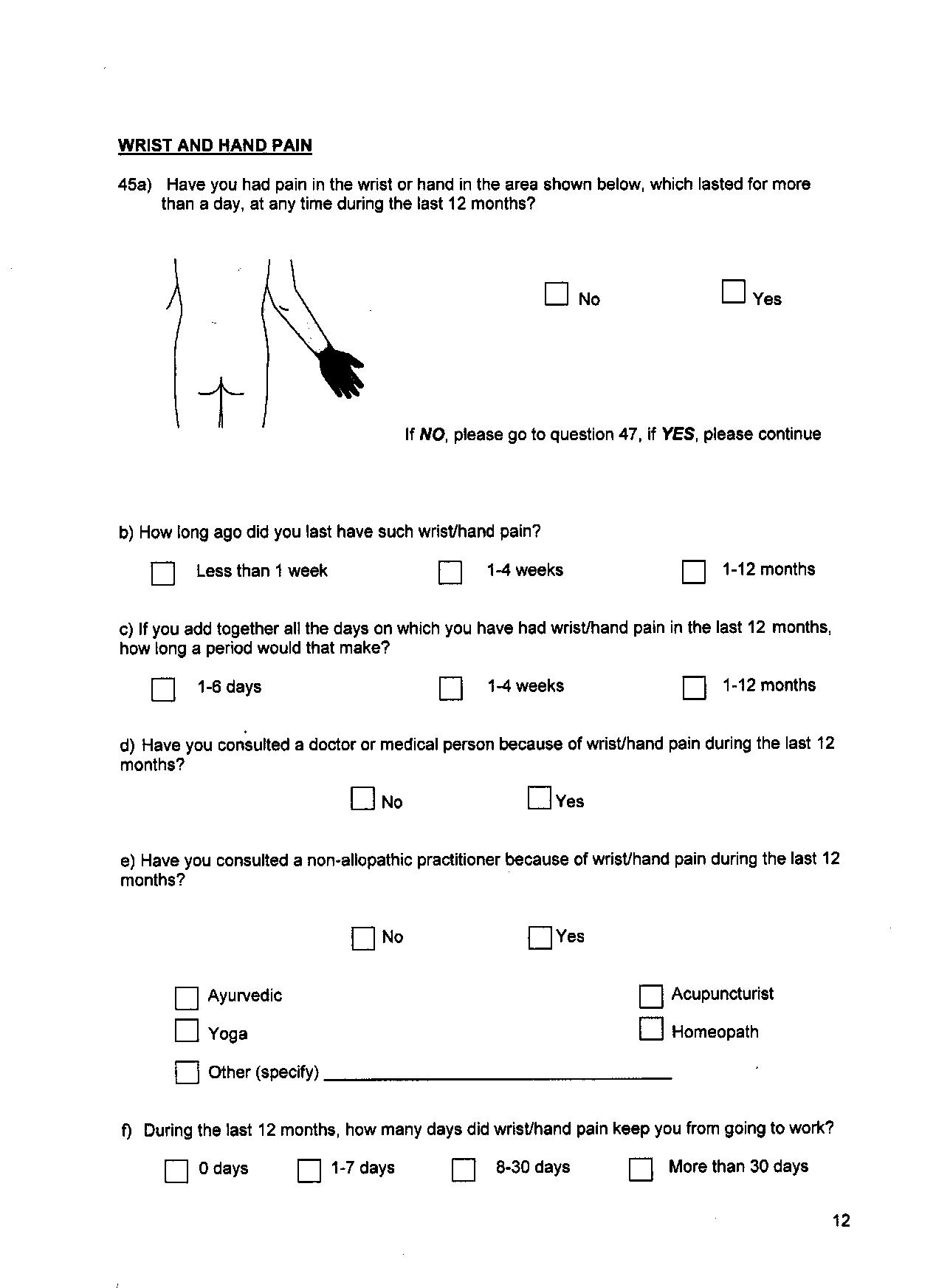


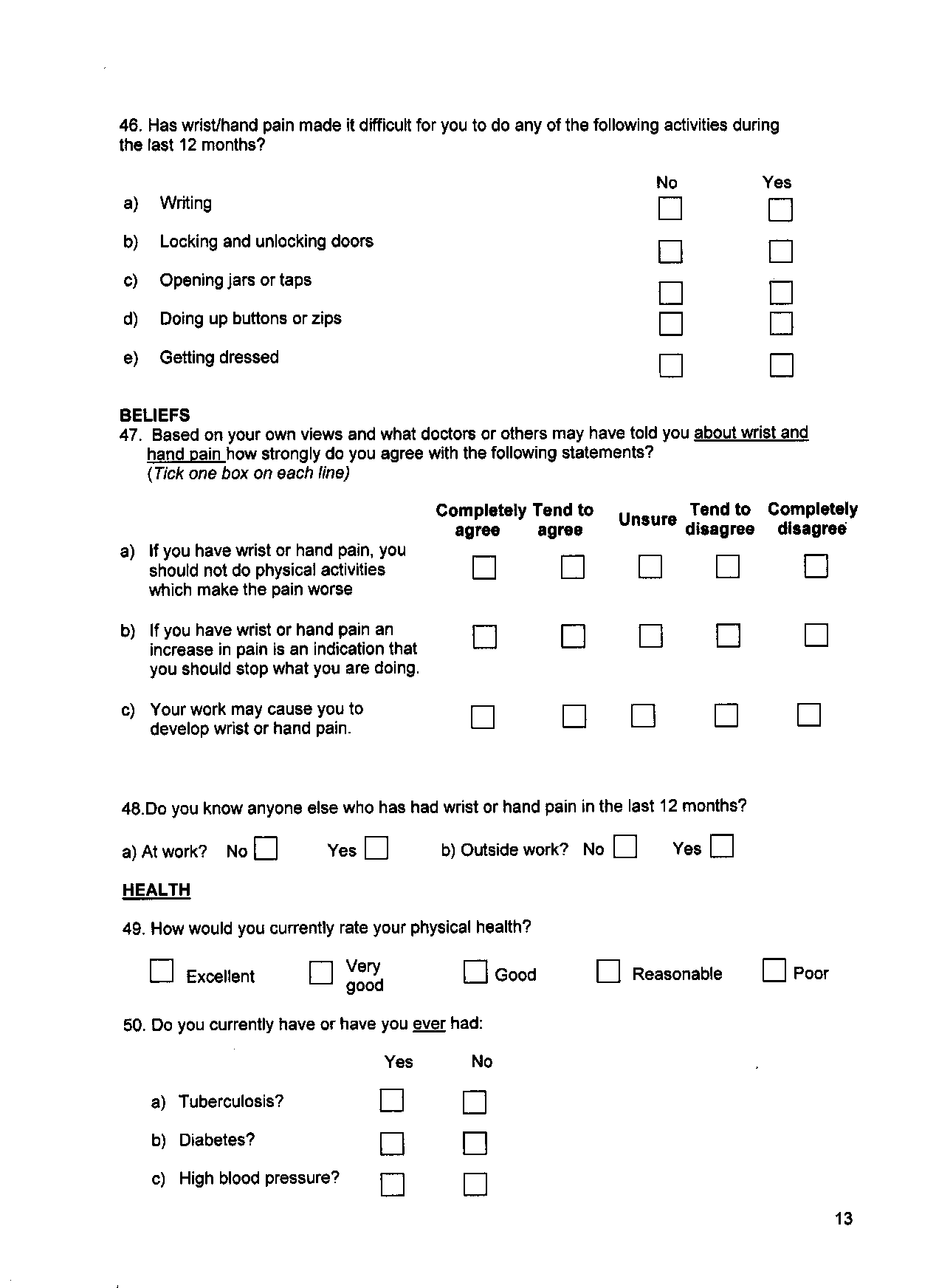


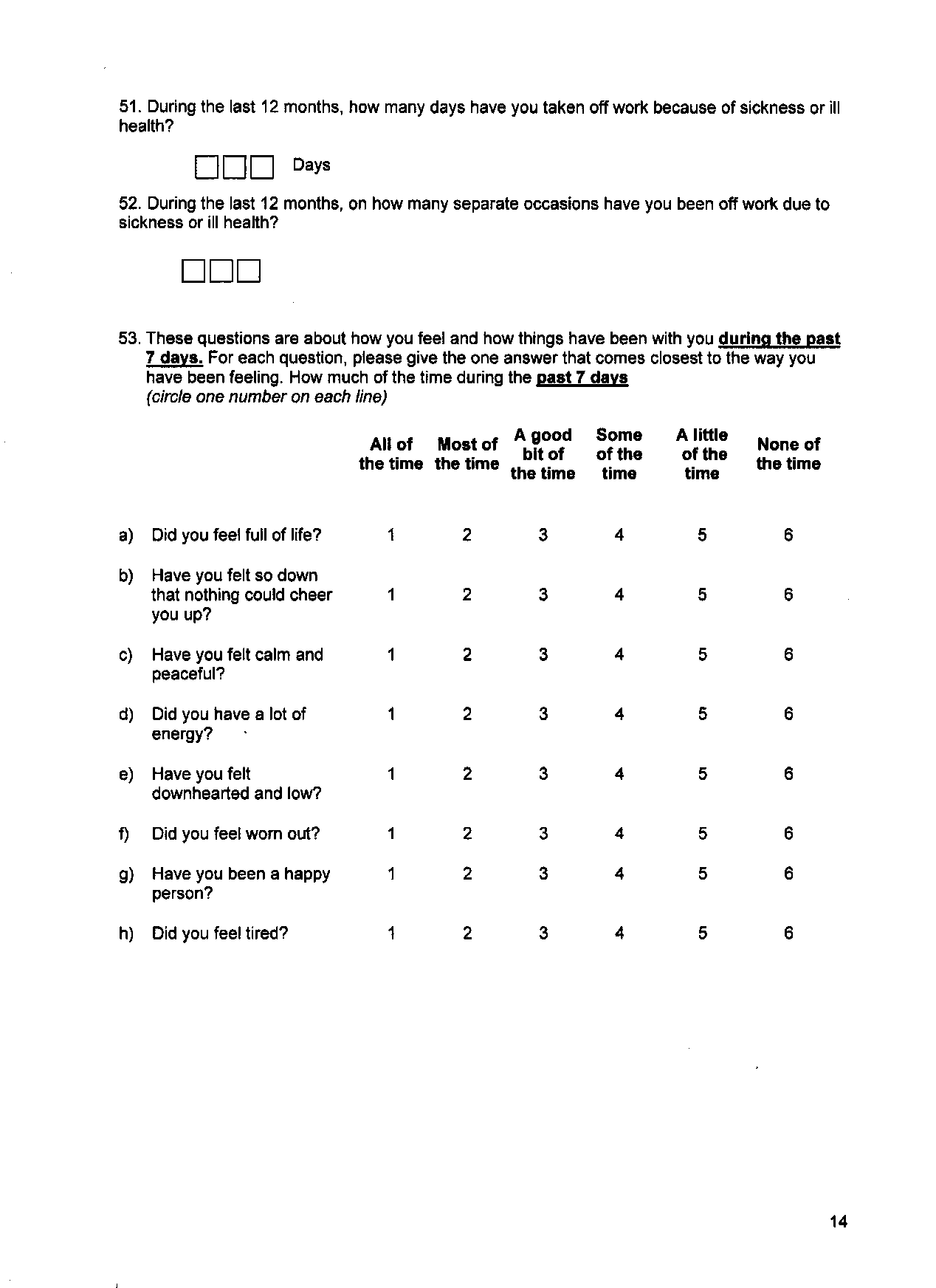


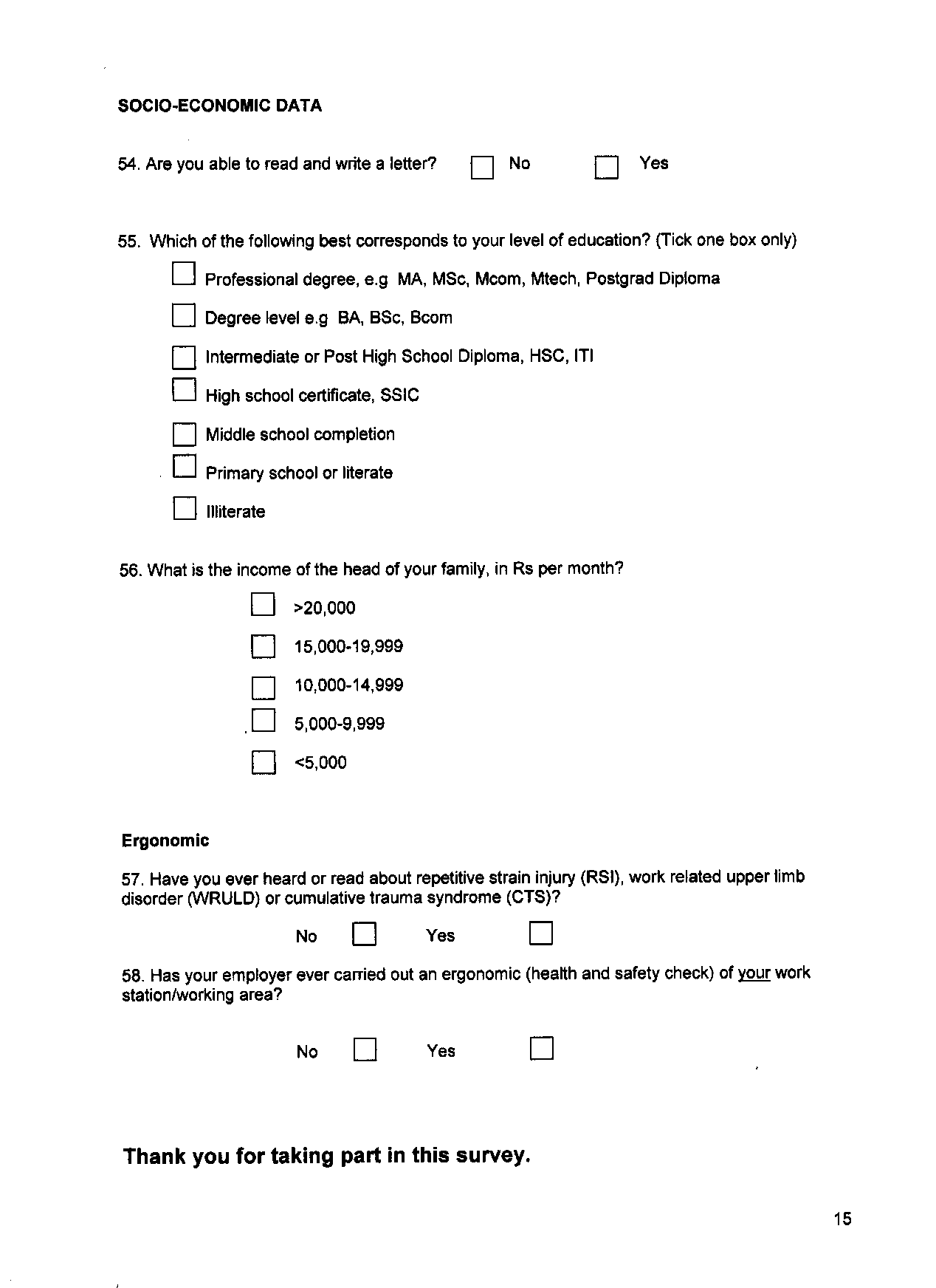

Supplement: Supplementary file 1 — India Questionnaire. Description of data: Copy of questionnaire used to collect data in India (DOCX 307 kb) [file 12891_2019_2494_MOESM1_ESM.docx]

**UK Questionnaire**


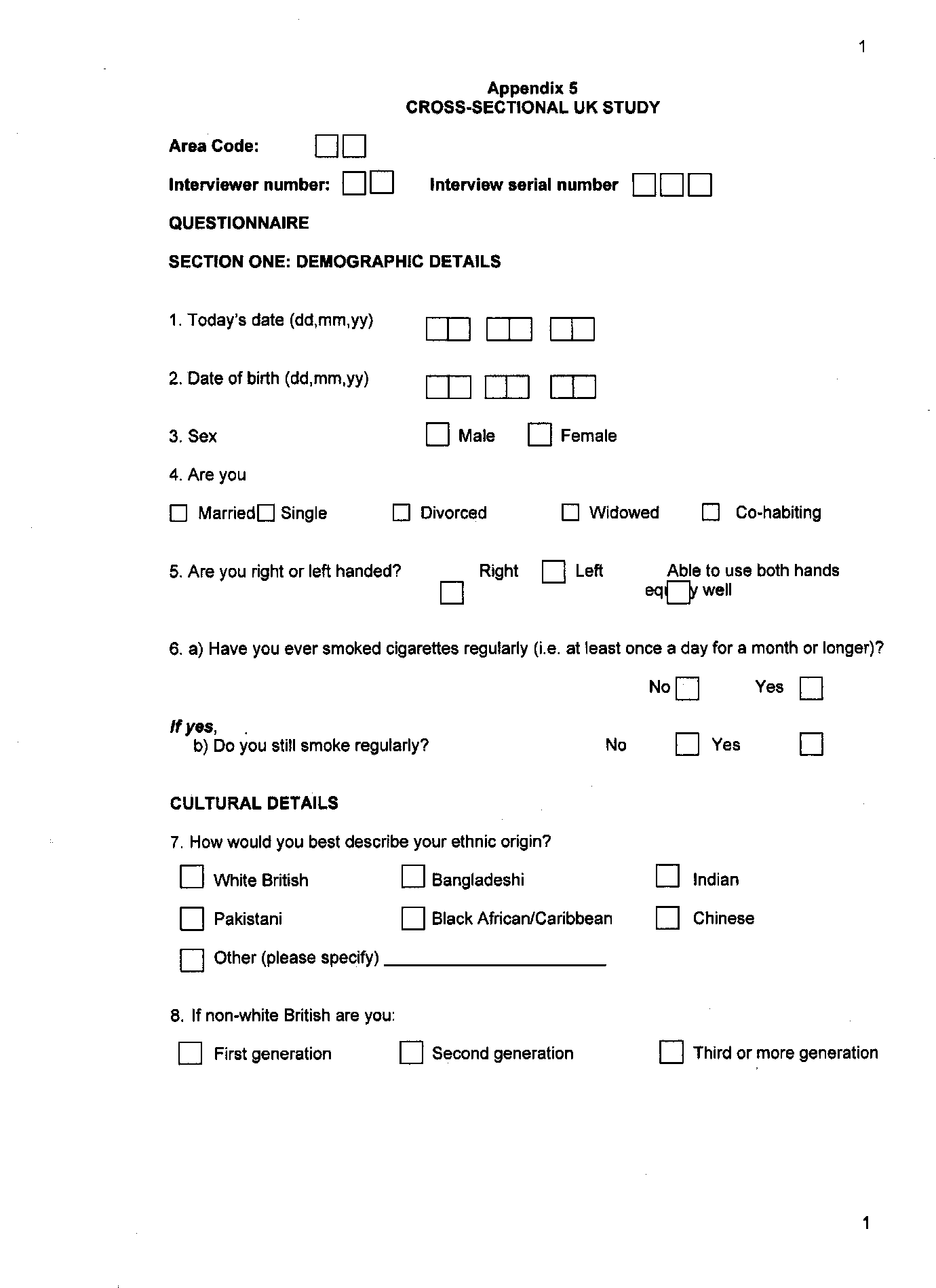


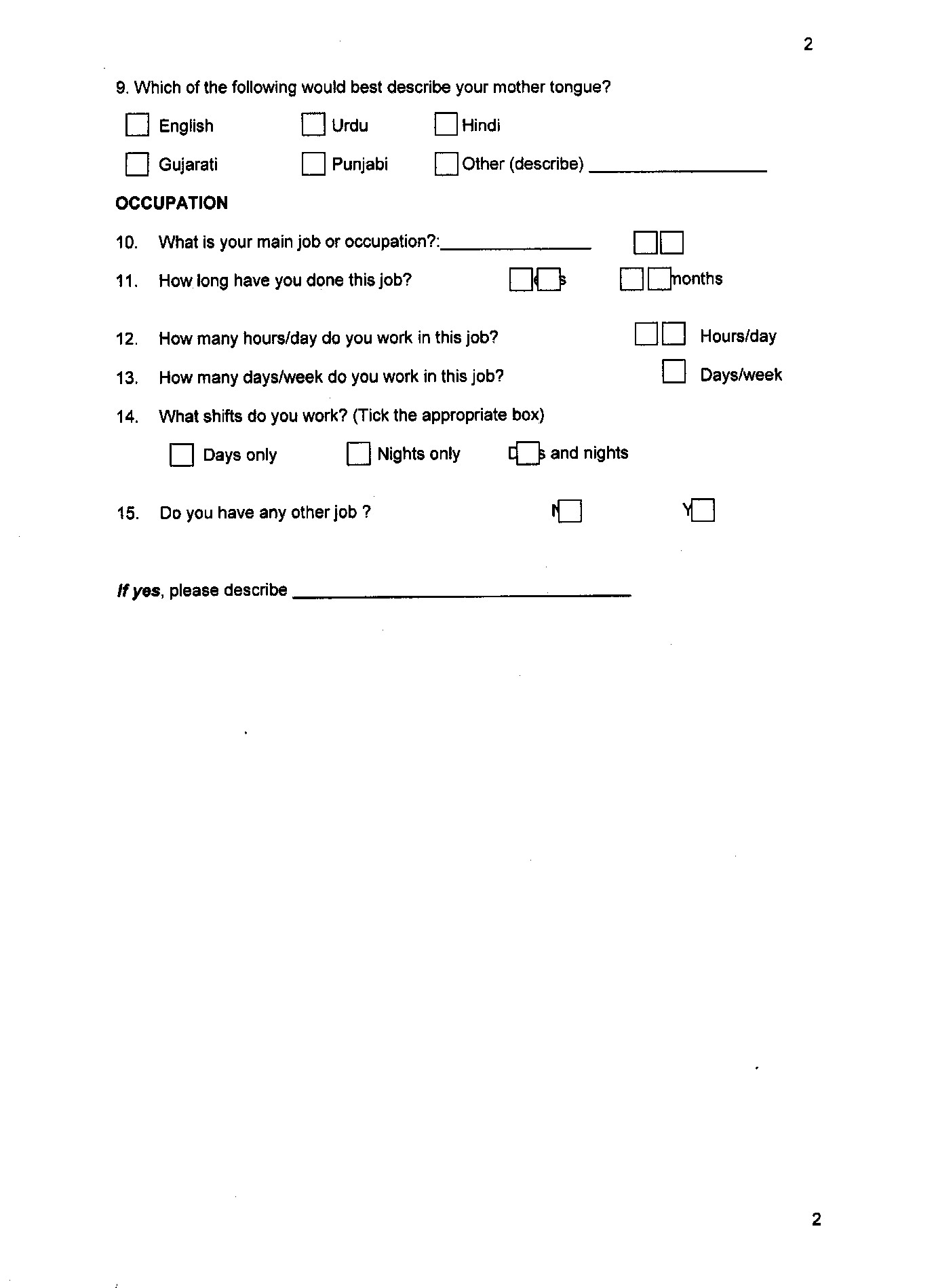


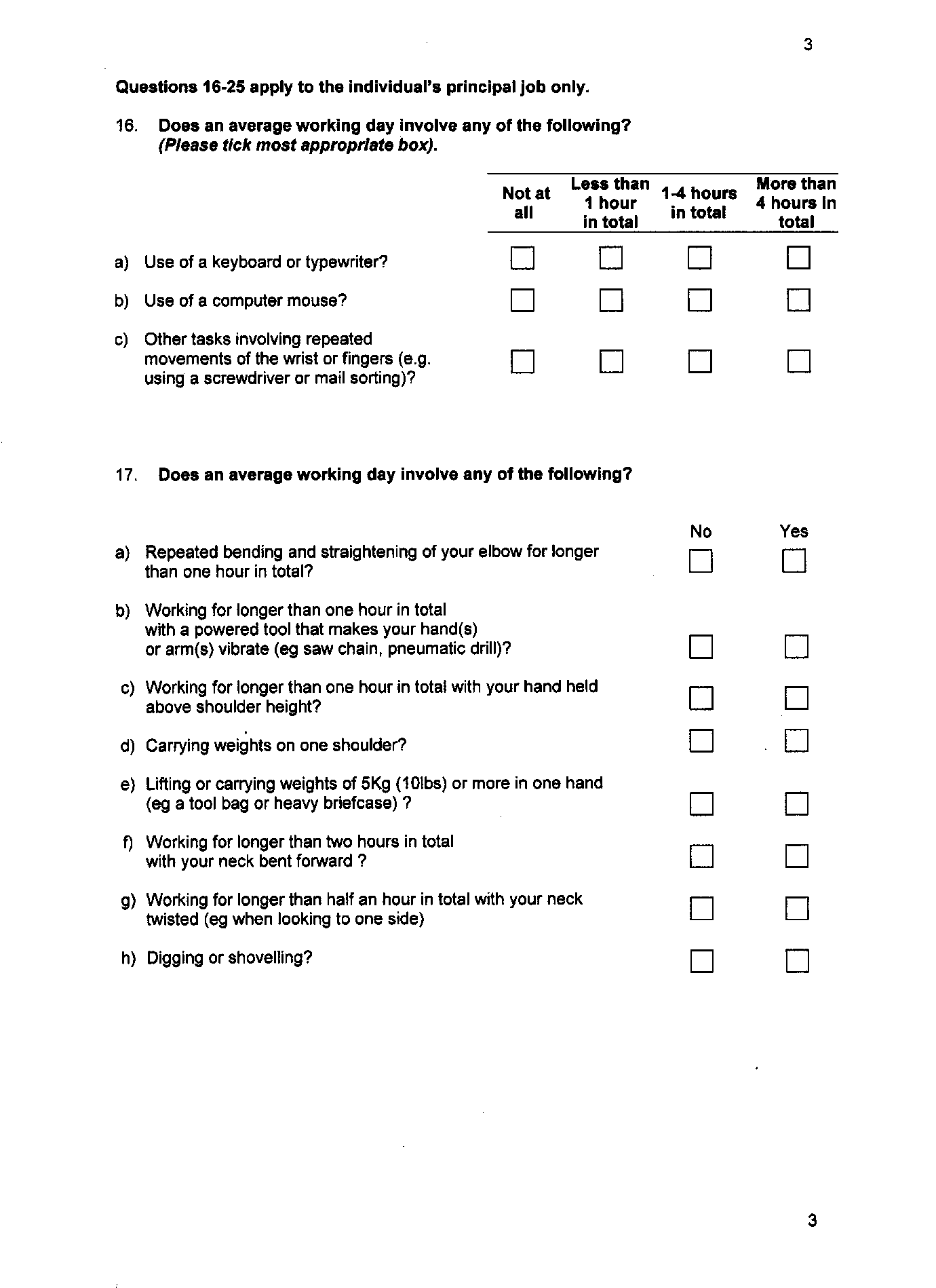


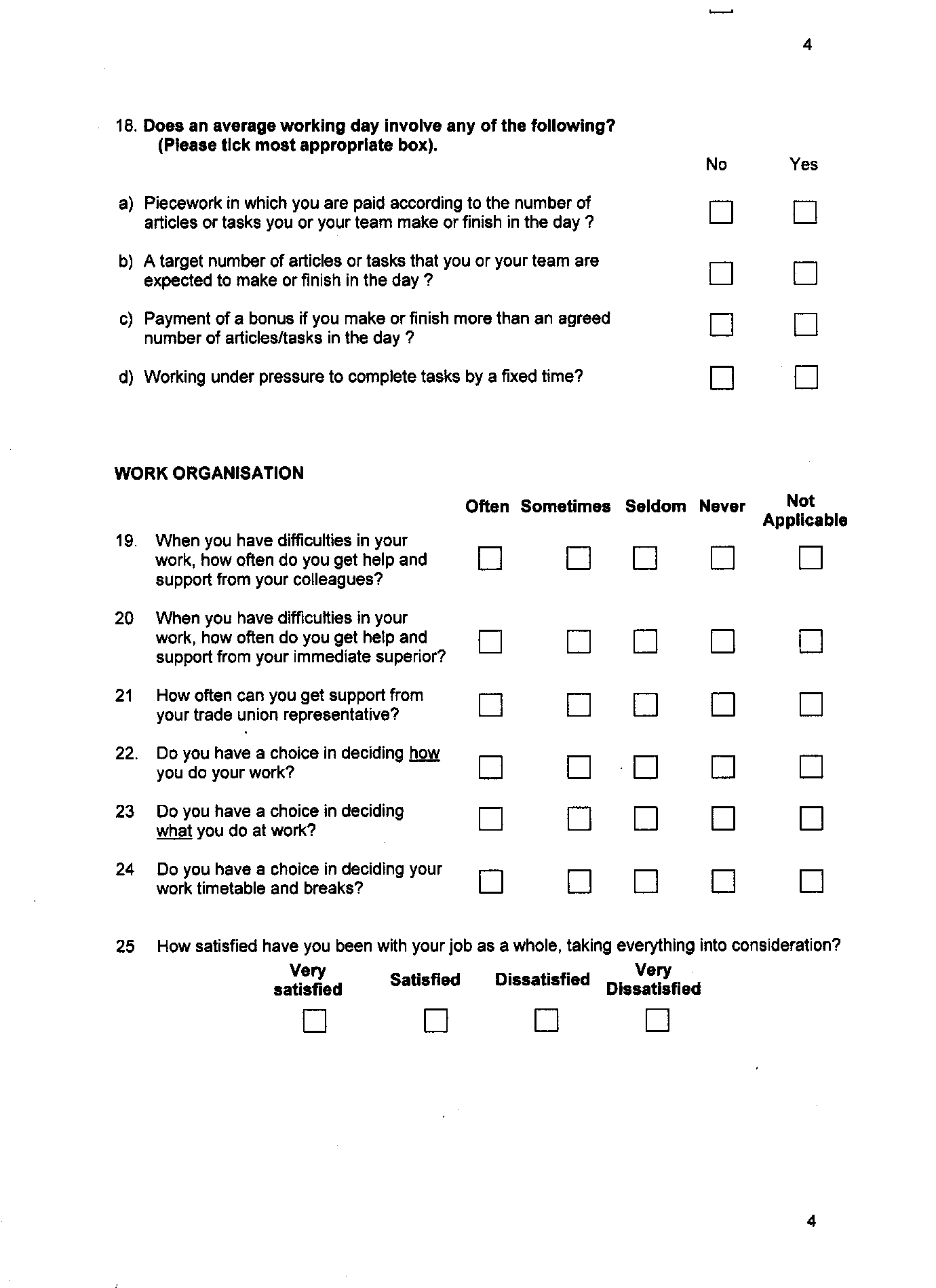


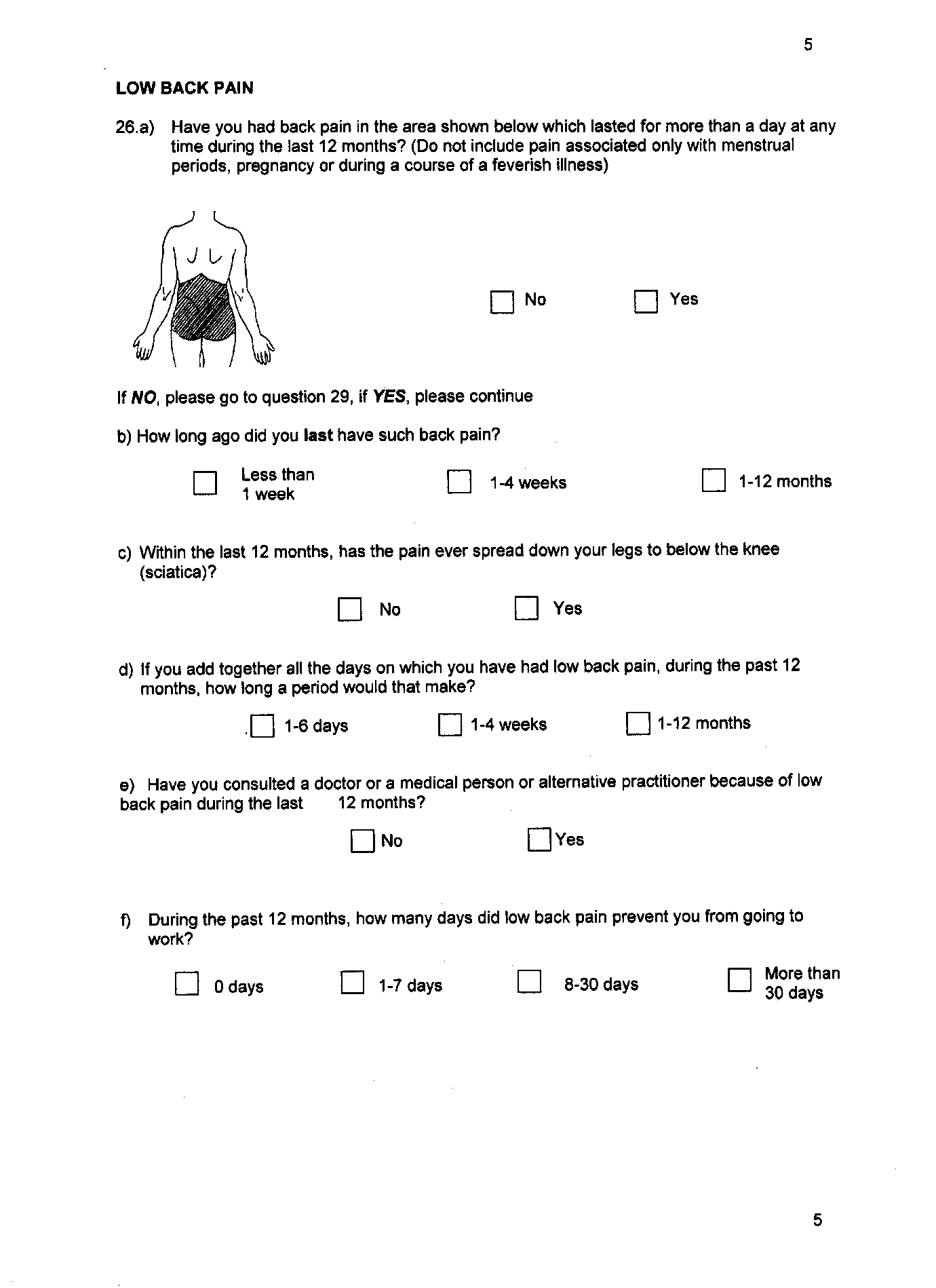


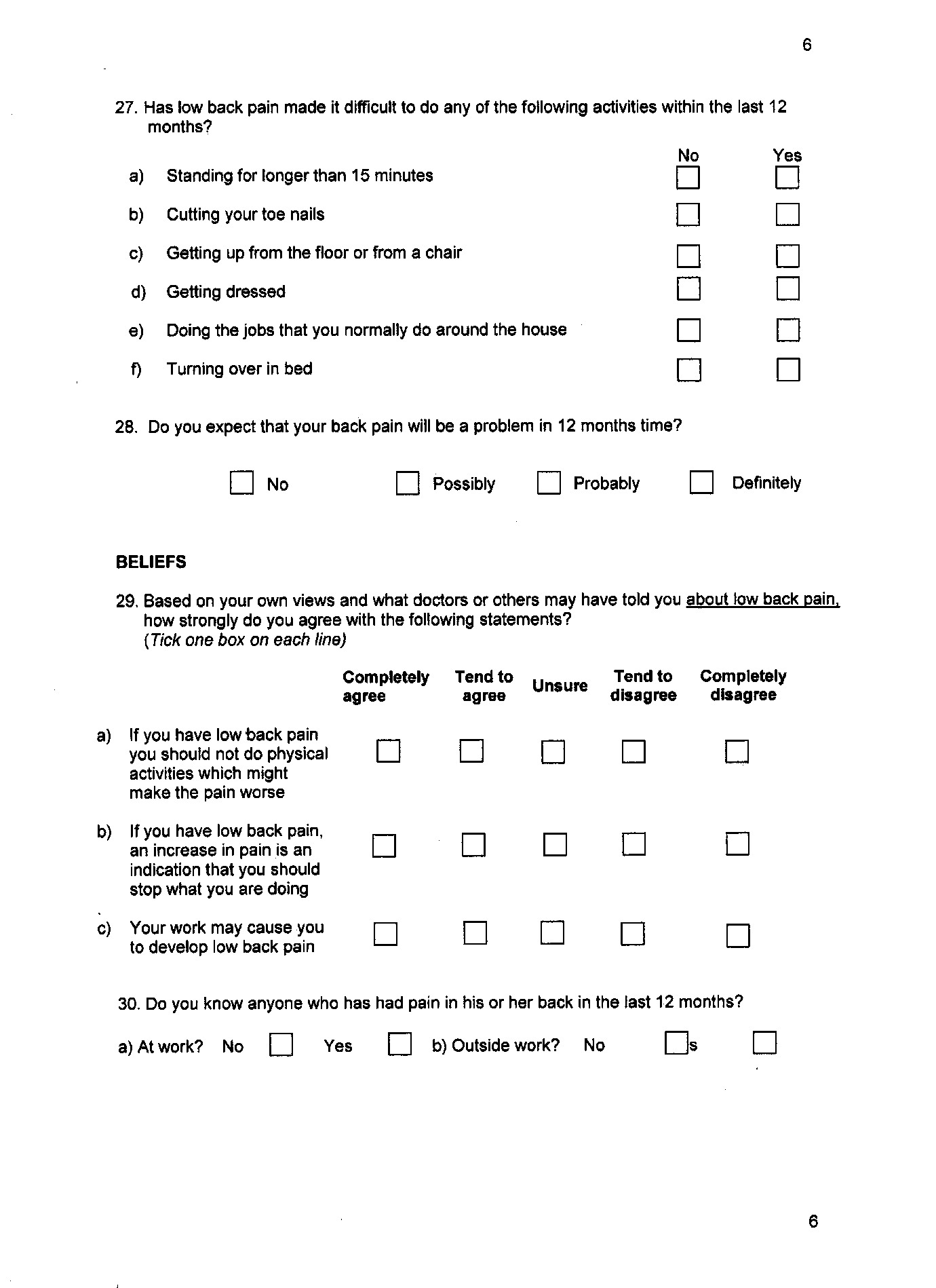


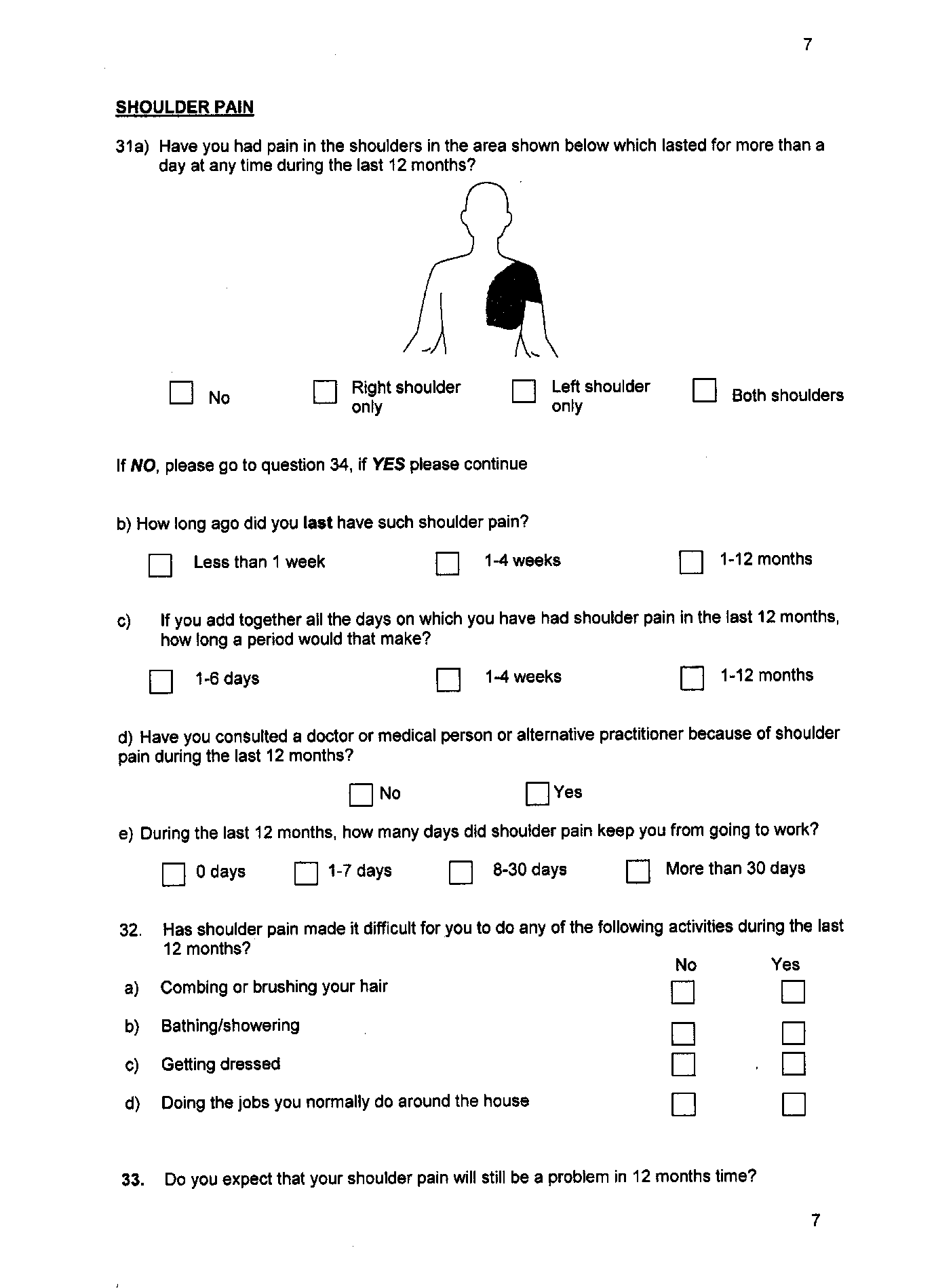


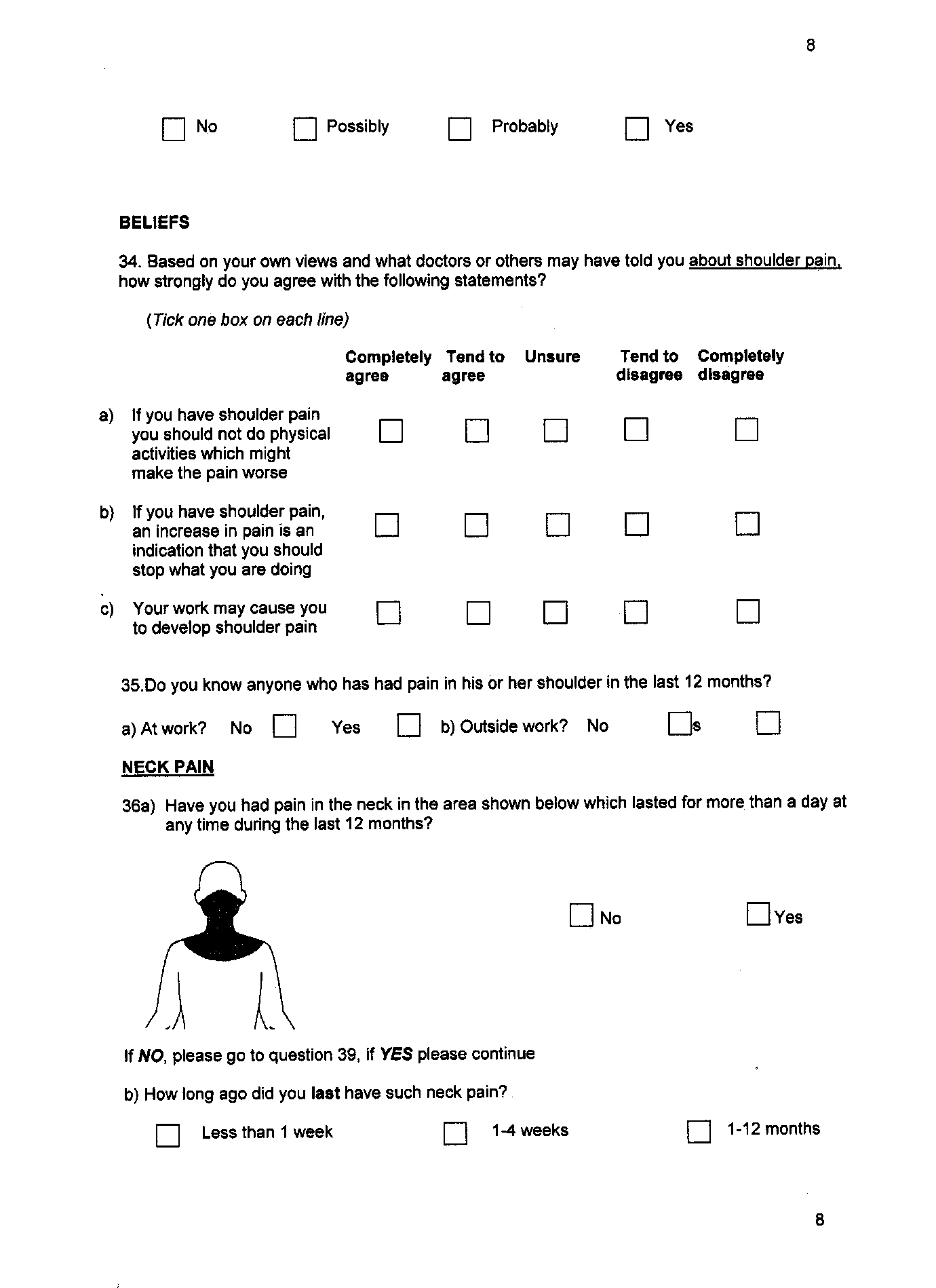


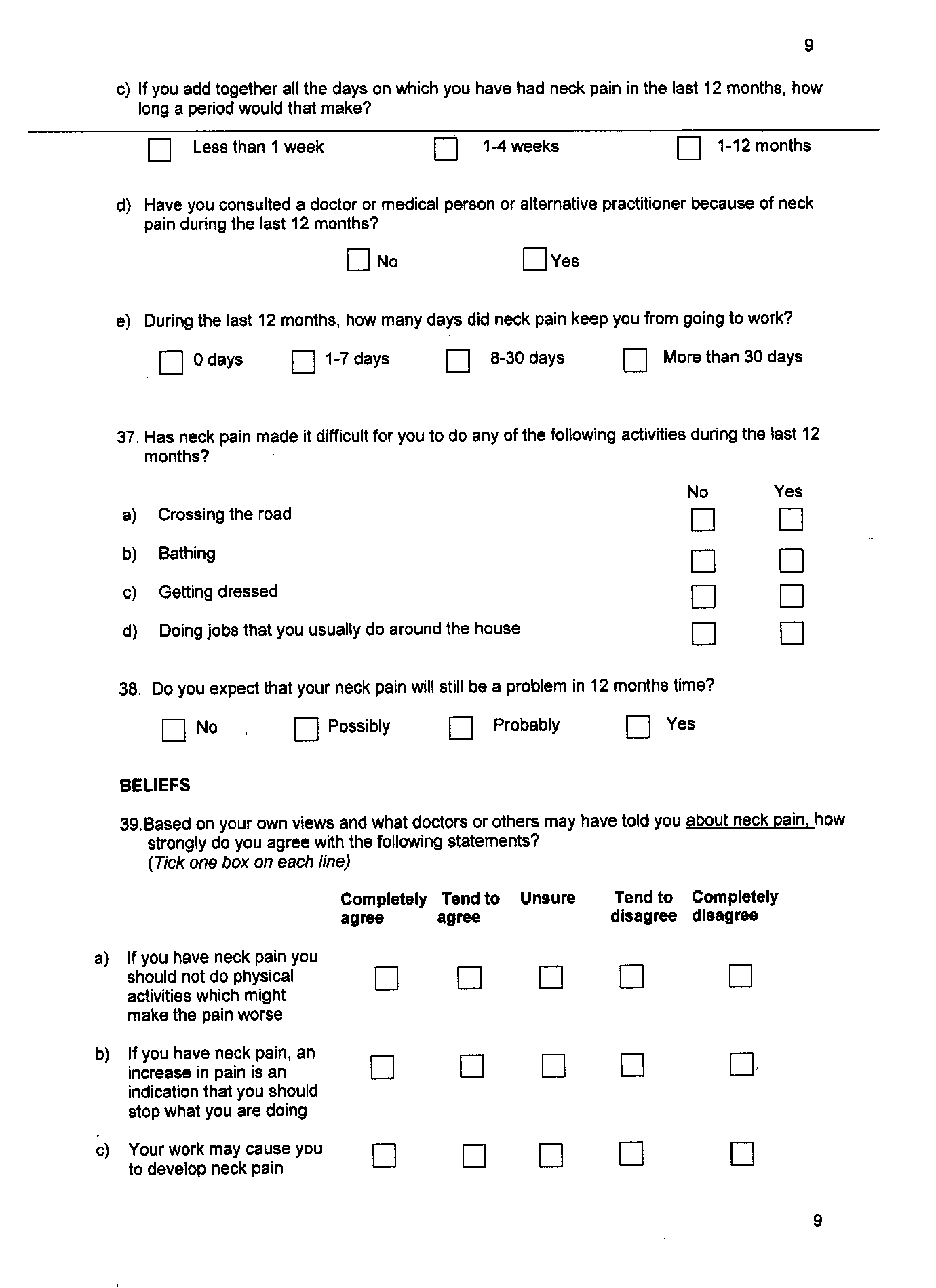


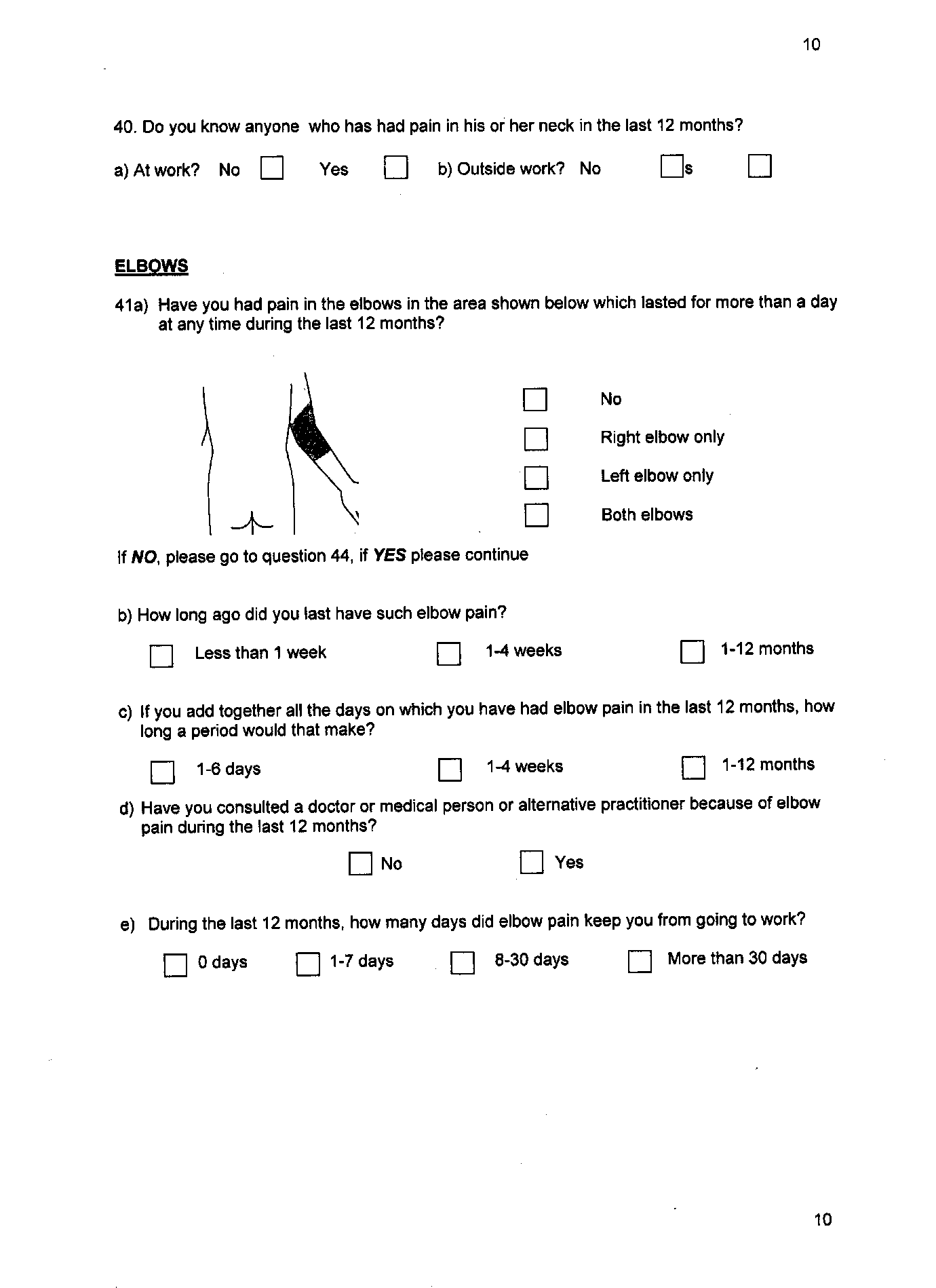


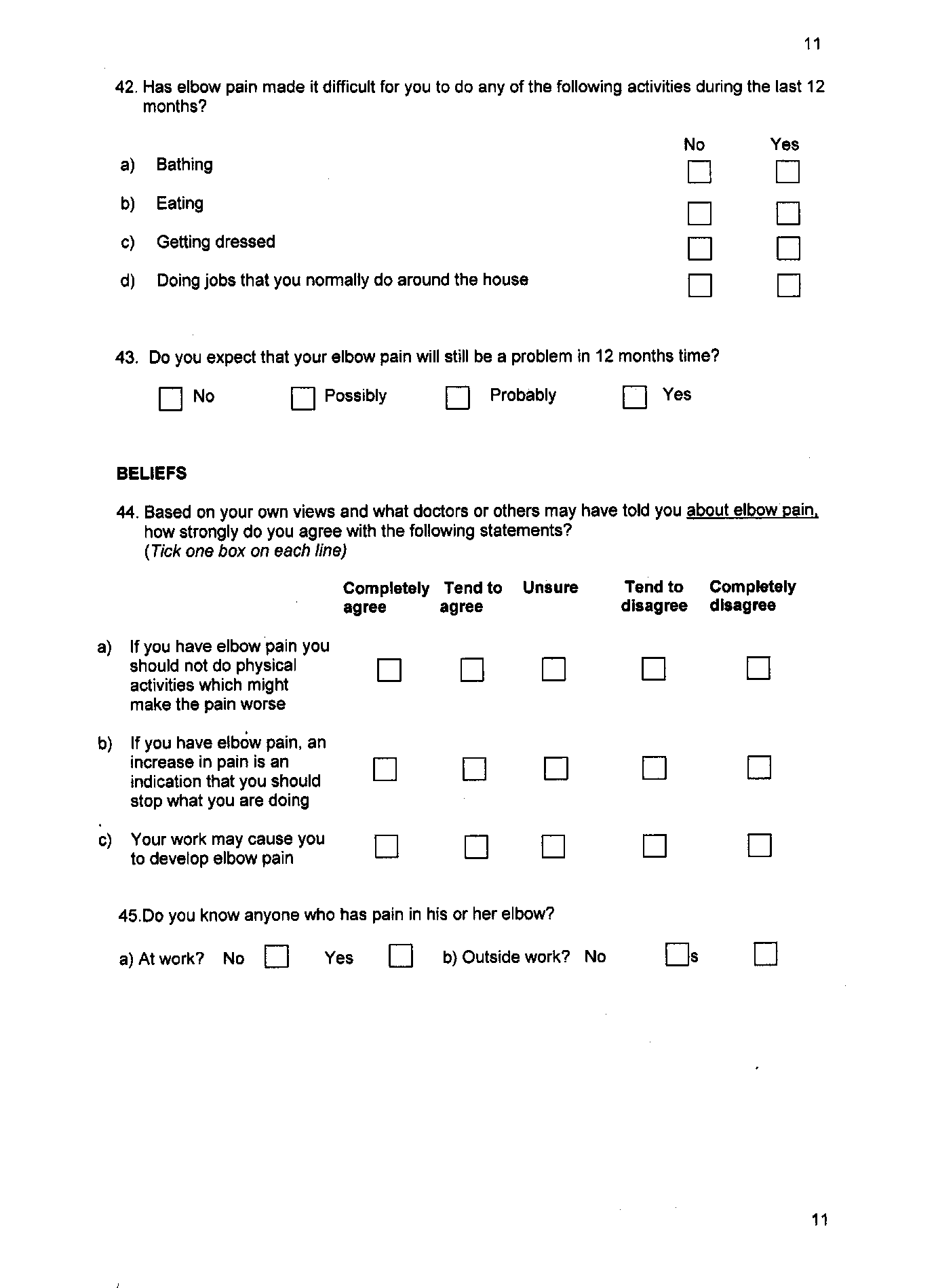


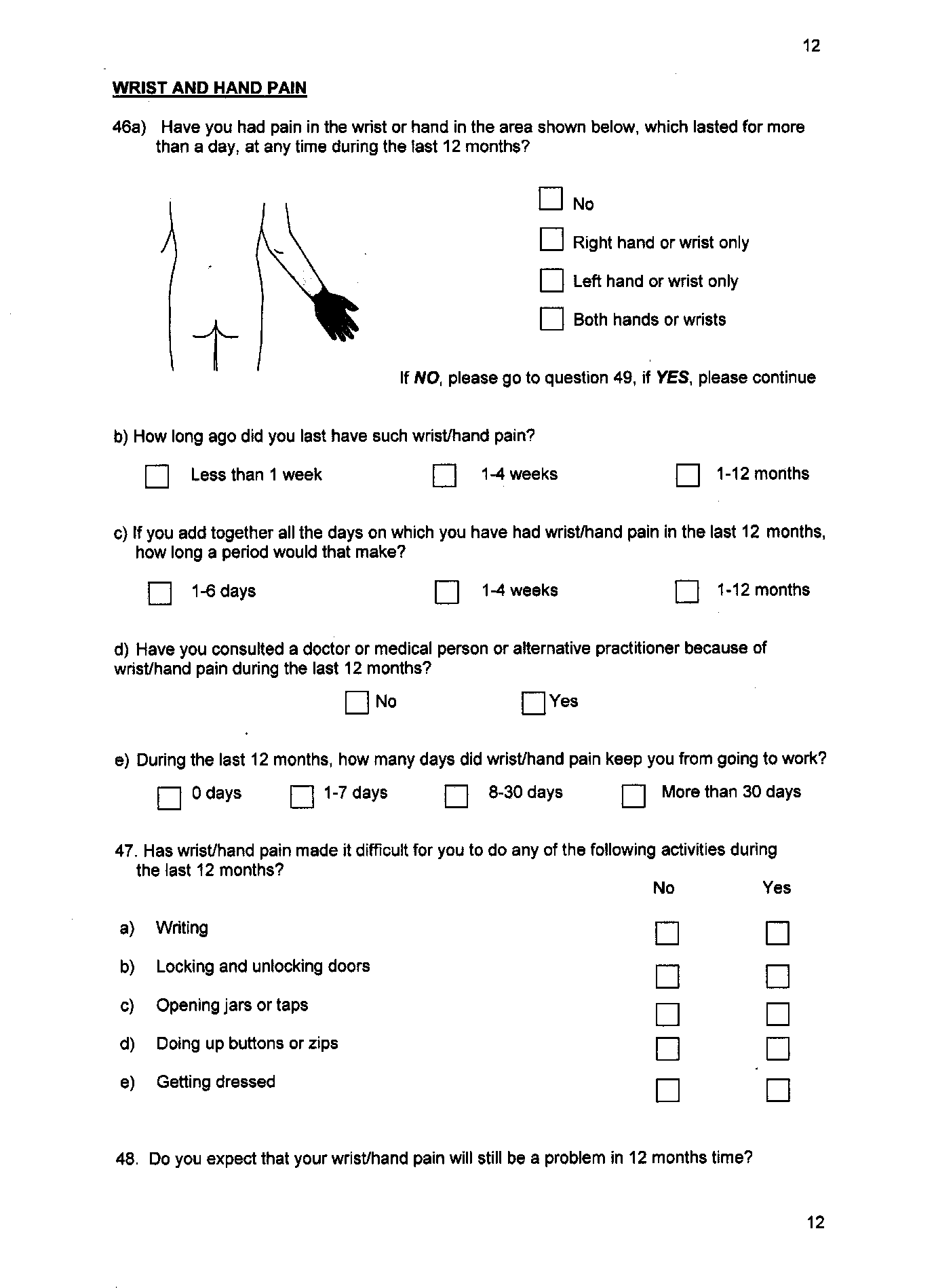


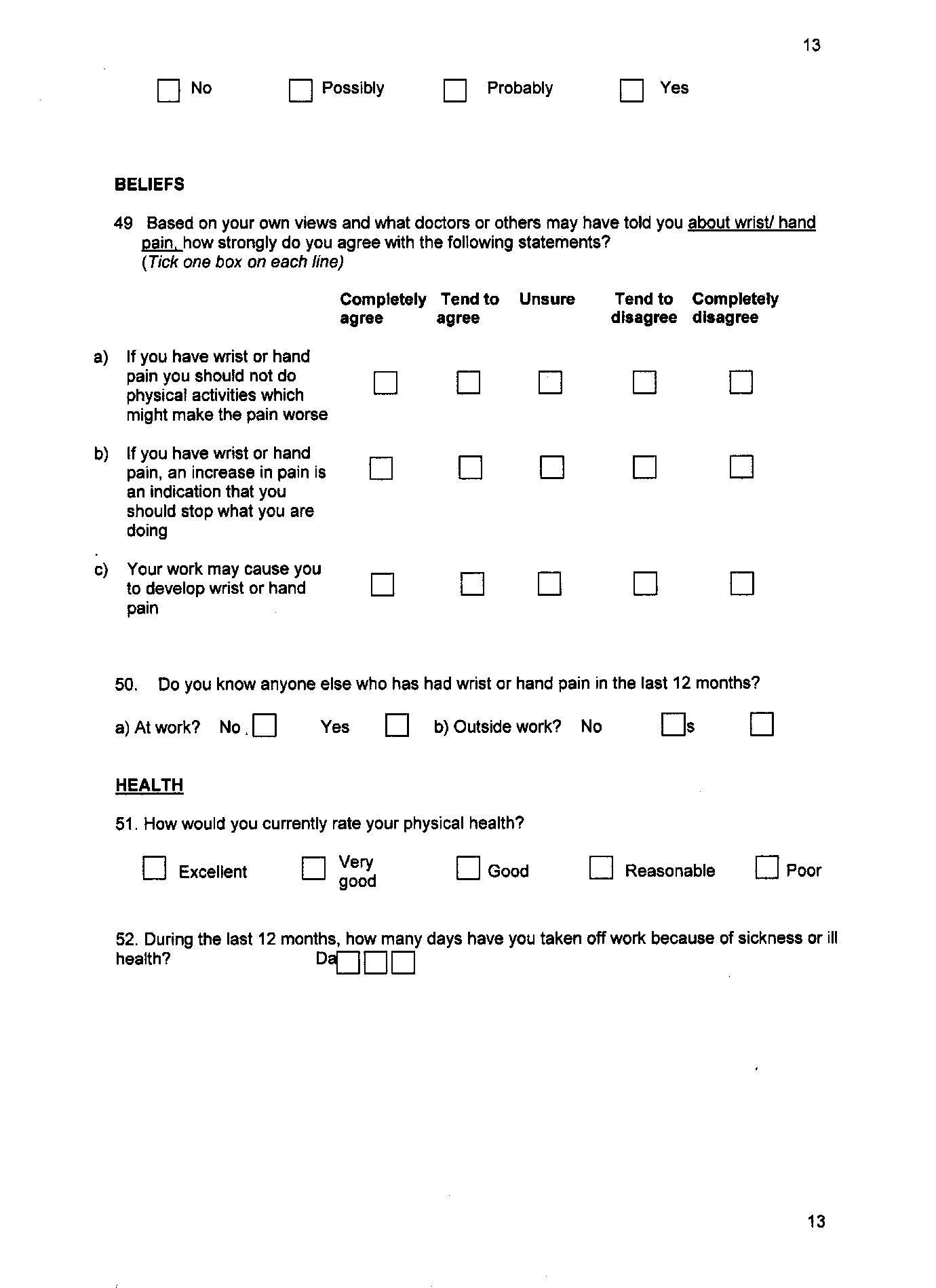


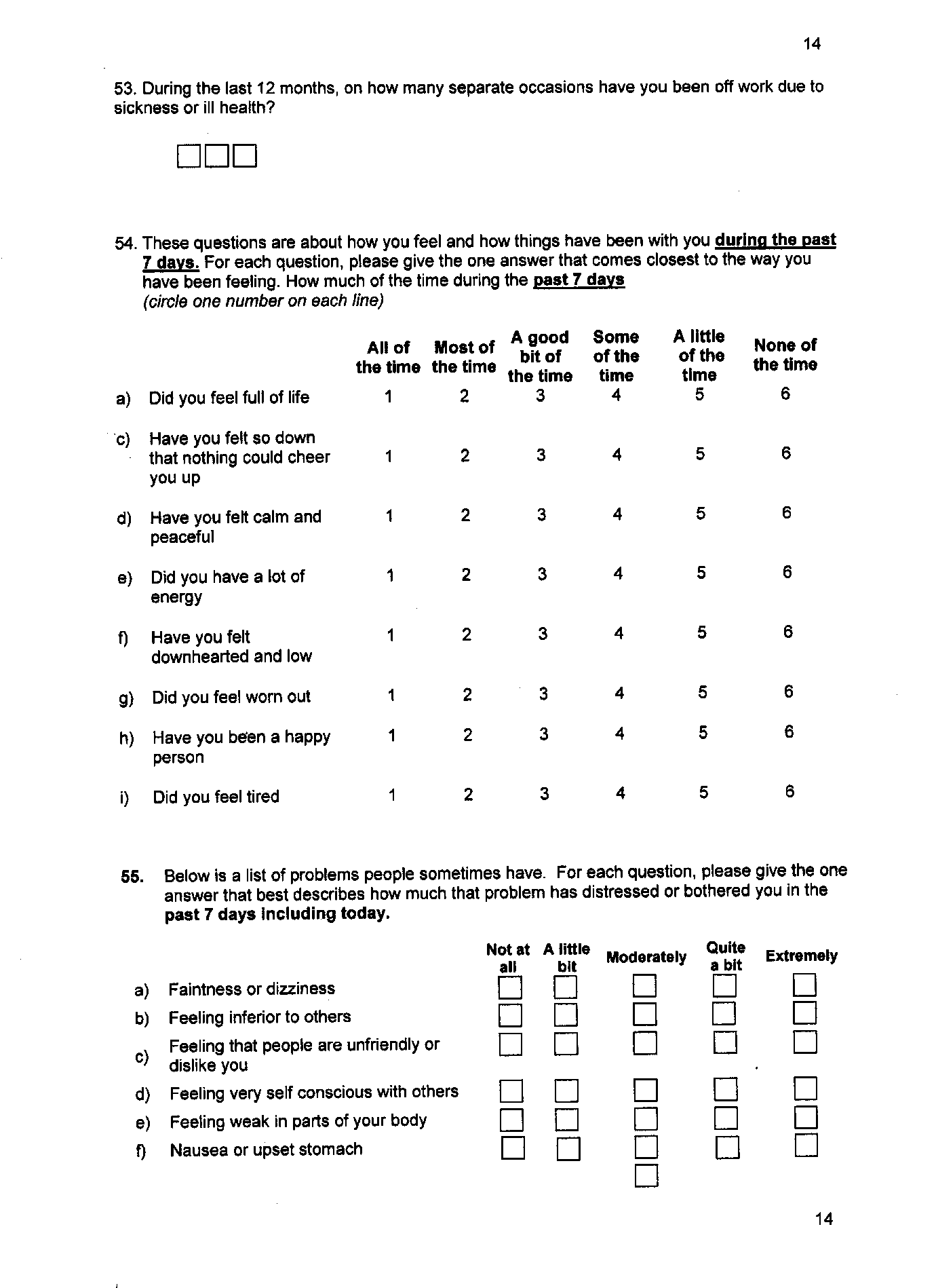


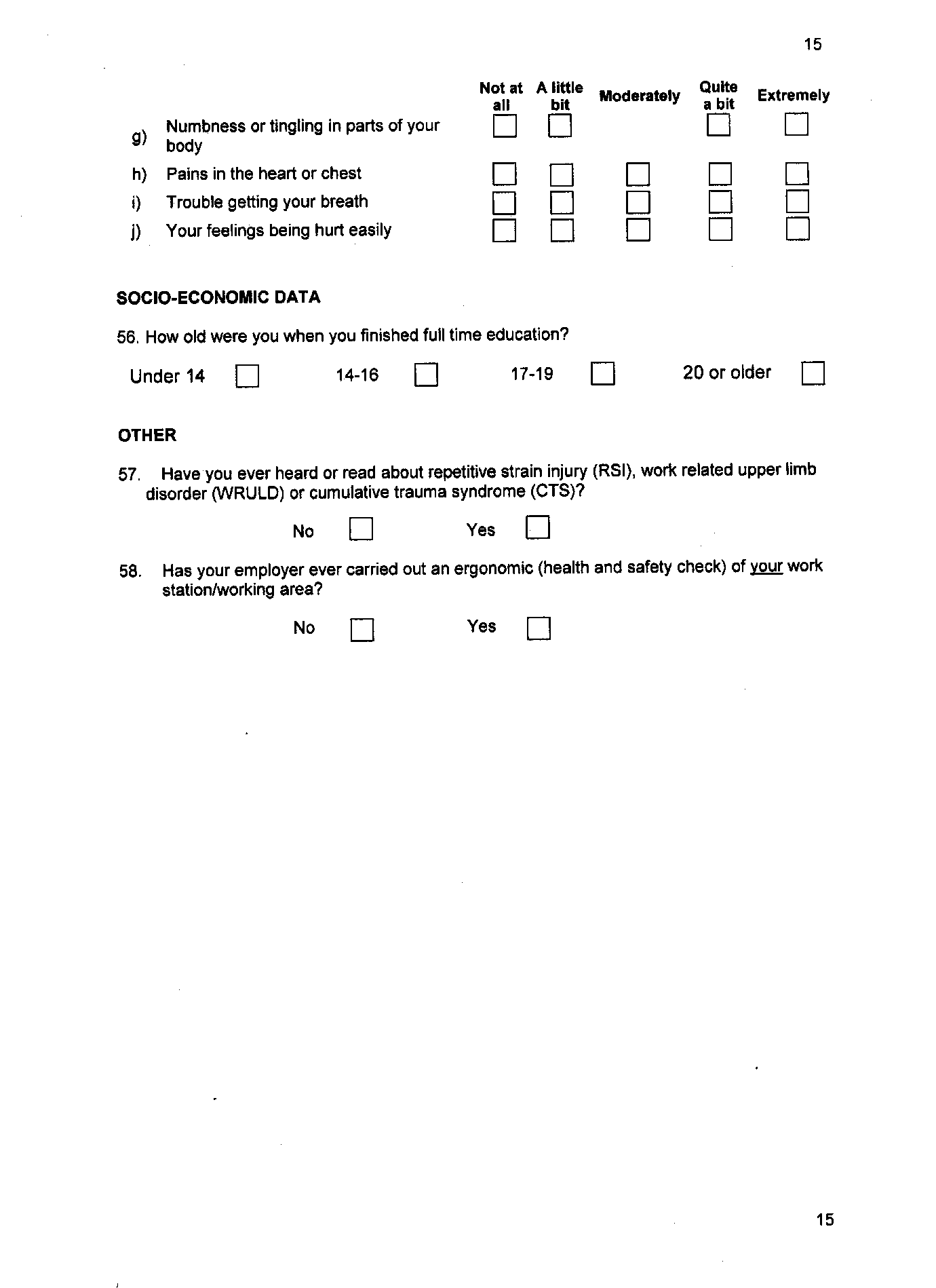

Supplement: Supplementary file 2 — UK Questionnaire. Description of data: Copy of questionnaire used to collect data in UK (DOCX 336 kb) [file 12891_2019_2494_MOESM2_ESM.docx]
